# Genomics and phenomics of body mass index reveals a complex disease network

**DOI:** 10.1038/s41467-022-35553-2

**Published:** 2022-12-29

**Authors:** Jie Huang, Jennifer E. Huffman, Yunfeng Huang, Ítalo Do Valle, Themistocles L. Assimes, Sridharan Raghavan, Benjamin F. Voight, Chang Liu, Albert-László Barabási, Rose D. L. Huang, Qin Hui, Xuan-Mai T. Nguyen, Yuk-Lam Ho, Luc Djousse, Julie A. Lynch, Marijana Vujkovic, Catherine Tcheandjieu, Hua Tang, Scott M. Damrauer, Peter D. Reaven, Donald Miller, Lawrence S. Phillips, Maggie C. Y. Ng, Mariaelisa Graff, Christopher A. Haiman, Ruth J. F. Loos, Kari E. North, Loic Yengo, George Davey Smith, Danish Saleheen, J. Michael Gaziano, Daniel J. Rader, Philip S. Tsao, Kelly Cho, Kyong-Mi Chang, Peter W. F. Wilson, VA Million Veteran Program, Yan V. Sun, Christopher J. O’Donnell

**Affiliations:** 1 School of Public Health and Emergency Management, Southern University of Science and Technology, Shenzhen, Guangdong China; 2 Center for Population Genomics, Massachusetts Veterans Epidemiology Research and Information Center (MAVERIC), VA Boston Healthcare System, Boston, MA USA; 3 Department of Epidemiology, Emory University Rollins School of Public Health, Atlanta, GA USA; 4 Atlanta VA Health Care System, Decatur, GA USA; 5 Network Science Institute and Department of Physics, Northeastern University, Boston, MA USA; 6 Division of Population Health and Data Science, MAVERIC, VA Boston Healthcare System, Boston, MA USA; 7 VA Palo Alto Health Care System, Palo Alto, CA USA; 8 Department of Medicine, Stanford University School of Medicine, Stanford, CA USA; 9 VA Eastern Colorado Healthcare System, Aurora, CO USA; 10 University of Colorado School of Medicine, Aurora, CO USA; 11 Corporal Michael J. Crescenz VA Medical Center, Philadelphia, PA USA; 12 Department of Systems Pharmacology and Translational Therapeutics, University of Pennsylvania, Philadelphia, PA USA; 13 Department of Genetics, University of Pennsylvania, Philadelphia, PA USA; 14 Institute of Translational Medicine and Therapeutics, University of Pennsylvania Perelman School of Medicine, Philadelphia, PA USA; 15 Channing Division of Network Medicine, Department of Medicine, Brigham and Women’s Hospital, Harvard Medical School, Boston, MA USA; 16 Department of Network and Data Science, Central European University, Budapest, Hungary; 17 Carle Illinois College of Medicine, Champaign, IL USA; 18 Massachusetts Veterans Epidemiology Research and Information Center (MAVERIC), VA Boston Healthcare System, Boston, MA USA; 19 Department of Medicine, Brigham and Women’s Hospital, Harvard Medical School, Boston, MA USA; 20 VA Salt Lake City Healthcare, Salt Lake City, UT USA; 21 University of Massachusetts, Boston, MA USA; 22 Department of Medicine; Perelman School of Medicine, University of Pennsylvania, Philadelphia, PA USA; 23 Department of Genetics, Stanford University School of Medicine, Stanford, CA USA; 24 Department of Surgery; Perelman School of Medicine, University of Pennsylvania, Philadelphia, PA USA; 25 Department of Genetics, University of Pennsylvania Perelman School of Medicine, Philadelphia, PA USA; 26 Phoenix VA Health Care System, Phoenix, AZ USA; 27 College of Medicine, University of Arizona, Phoenix, AZ USA; 28 Center for Healthcare Organization and Implementation Research, Bedford VA Medical Center, Bedford, MA USA; 29 Division of Endocrinology, Department of Medicine, Emory University School of Medicine, Atlanta, GA USA; 30 Vanderbilt Genetics Institute and Division of Genetic Medicine, Vanderbilt University Medical Center, Nashville, TN USA; 31 Gillings School of Global Public Health, Department of Epidemiology, University of North Carolina Chapel Hill, Chapel Hill, NC USA; 32 Department of Preventive Medicine, Keck School of Medicine, University of Southern California, Los Angeles, CA USA; 33 The Charles Bronfman Institute for Personalized Medicine, Icahn School of Medicine at Mount Sinai, New York, NY USA; 34 The Mindich Child Health and Development Institute, Icahn School of Medicine at Mount Sinai, New York, NY USA; 35 Novo Nordisk Foundation Center for Basic Metabolic Research, Faculty of Health and Medical Sciences, University of Copenhagen, Copenhagen, Denmark; 36 Institute for Molecular Bioscience, The University of Queensland, Brisbane, Australia; 37 Population Health Sciences, Bristol Medical School, University of Bristol, Bristol, UK; 38 Medical Research Council Integrative Epidemiology Unit, University of Bristol, Bristol, UK; 39 Center for Non-Communicable Diseases, Karachi, Sindh Pakistan; 40 Department of Medicine, Columbia University Irving Medical Center, New York, NY USA; 41 Department of Cardiology, Columbia University Irving Medical Center, New York, NY USA; 42 Division of Cardiology, Emory University School of Medicine, Atlanta, Georgia USA; 43 Cardiology Section, VA Boston Healthcare System, Boston, MA USA

**Keywords:** Obesity, Genetic variation

## Abstract

Elevated body mass index (BMI) is heritable and associated with many health conditions that impact morbidity and mortality. The study of the genetic association of BMI across a broad range of common disease conditions offers the opportunity to extend current knowledge regarding the breadth and depth of adiposity-related diseases. We identify 906 (364 novel) and 41 (6 novel) genome-wide significant loci for BMI among participants of European (N~1.1 million) and African (N~100,000) ancestry, respectively. Using a BMI genetic risk score including 2446 variants, 316 diagnoses are associated in the Million Veteran Program, with 96.5% showing increased risk. A co-morbidity network analysis reveals seven disease communities containing multiple interconnected diseases associated with BMI as well as extensive connections across communities. Mendelian randomization analysis confirms numerous phenotypes across a breadth of organ systems, including conditions of the circulatory (heart failure, ischemic heart disease, atrial fibrillation), genitourinary (chronic renal failure), respiratory (respiratory failure, asthma), musculoskeletal and dermatologic systems that are deeply interconnected within and across the disease communities. This work shows that the complex genetic architecture of BMI associates with a broad range of major health conditions, supporting the need for comprehensive approaches to prevent and treat obesity.

## Abstract (web summary)

Elevated body mass index is heritable and associated with many health conditions that impact morbidity and mortality. Here, the authors identify greater than 900 genetic loci for body mass index (BMI) and find over 300 diagnoses associated with increasing BMI.

## Introduction

The global prevalence and disease burden of obesity continues to rise in the United States and worldwide^1,2^, posing a major threat to public health and quality of life. Variation in body mass index (BMI), the metric commonly used to define obesity, is highly heritable. Obesity and extreme obesity is strongly associated with a growing number of chronic diseases, including type 2 diabetes mellitus (T2DM) and coronary heart disease, that are leading causes of preventable morbidity and mortality. It remains uncertain whether obesity lies in the causal pathway, is a confounding factor, or shares a common etiology with these co-occurring disease conditions. The totality of trials of metabolic bariatric surgery provides consistent efficacy for weight loss and growing evidence of increased survival and other health benefits associated with surgery^3–5^. Randomized clinical trials of pharmacotherapy with newer glucose-lowering agents, including SGLT2 inhibitors and GLP-1 receptor agonists, has also shown evidence of weight loss associated with reduced risk of major cardiovascular outcomes in patients with type 2 diabetes^6^, and treatment with GLP-1 receptor agonists plus lifestyle interventions is associated with sustained weight loss in obese nondiabetics^7^, although further study is needed to assess tolerability and overall clinical benefits^8^. Nevertheless, data are sparse on the interconnectedness of BMI-related conditions that could be influenced by emerging surgical and pharmacological approaches to obesity prevention and treatment. Mendelian randomization (MR)^9^ provides estimates of the strength of associations with disease outcomes using genetic instruments for life-course exposure to BMI, providing an approach to investigate the potential causal role of BMI in cardiometabolic disease^10^ and many other common diseases^11–13^.

A recent meta-analysis of genome-wide association studies (GWAS) for BMI identified over 700 independent variants in European descent populations^14^, implicating a large number of genes and pathways regulating satiety, energy balance and metabolism in adipose tissue. Genetic risk scores (GRS) based on BMI genetic variants robustly predict BMI and have enabled their use as genetic instruments in MR studies to address the relationship between BMI and several individual clinical disorders^10,12,15^. Phenome-wide association studies (PheWAS) offer a complementary framework to investigate genetic associations across many common diseases simultaneously^13,16^. While there is strong and consistent genetic evidence for the association of BMI with a number of leading causes of death^17^, the comprehensive assessment for MR associations within a PheWAS in a large population may expand our knowledge of the breadth, depth and interconnectedness of conditions associated with obesity.

We report here thousands of genome-wide single nucleotide polymorphism (SNP) associations with BMI in both European (EA) and African (AA) descent populations in the Million Veteran Program (MVP) mega-biobank, including meta-analysis with other large-scale multi-ancestry consortia and UK Biobank. We harness the breadth of BMI genetic variation and medical disease phenotypes in the Veterans Health Administration electronic health record (EHR) to conduct a phenome-wide MR study of the association of increased BMI with an extensive range of conditions accounting for a substantial burden of morbidity and mortality in the population.

## Results

### Observational analysis of the MVP participants

Among 215,734 EA (93.0% male) and 55,525 AA (87.6% male) MVP participants, the mean (SD) age at enrollment was 64.0 (13.1) and 57.9 (12.0) years, the mean (SD) BMI was 29.9 (5.9) and 29.2 (4.8) kg/m^2^, and the prevalence of obesity (BMI≥30) was 44.2% and 42.9%, respectively. Descriptive statistics of the EA and AA participants are summarized in Table 1.

**Table 1:** Characteristics of the Million Veteran Program non-Hispanic EA and AA participants

	EA (*N* = 215,734) Mean ± SD	AA *(N* = 55,525) Mean ± SD
Male gender *n* (%)	200,733 (93.0%)	48,628 (87.6%)
Age at Enrollment (years) mean ± SD	63.95 ± 13.11	57.87 ± 11.96
BMI (kg/m^2^) mean ± SD	29.88 ± 5.86	29.15 ± 4.78
Overweight (30>BMI≥25), *n* (%)	81,382 (37.7%)	20,471 (36.9%)
Obesity (BMI ≥ 30), *n* (%)	95,277 (44.2%)	23,794 (42.9%)
maxTC (mg/dL) mean ± SD	224.60 ± 47.95	226.61 ± 48.71
Statin use at enrollment	128,075 (59.4%)	28,854 (52.0%)
CHD^a^, *n* (%)	64,597 (29.9%)	12,652 (22.8%)
PAD^a^, *n* (%)	16,331 (7.6%)	3503 (6.3%)

*EA* European American, *AA* African American, *BMI* body mass index, *SD* standard deviation, *IQR* Interquartile Range, *max* maximal level among multiple longitudinal measures, *TC* Total Cholesterol, *CHD* coronary heart disease, *PAD* peripheral artery disease.

^a^defined using inpatient and outpatient ICD-9 and ICD-10 codes available in EHR data at enrollment.

### GWAS and genetic instruments in European and African Americans

The genetic ancestries of the MVP non-Hispanic EAs and AAs were consistent with the population structure and admixture represented by the top principal components (Supplementary Fig. 1). The major analytical procedures including the GWAS, PheWAS and network analyses are outlined in Supplementary Fig. 2. For EA participants, we identified 795 genome-wide significant loci (lead SNPs *p* < 5×10^−8^) in MVP, of which 285 were novel (distance > 500 kb and LD r^2^ < 0.1) compared with previously reported BMI-associated loci^14^. In the combined meta-analysis of MVP, UK Biobank and the GIANT Consortium, including up to 1,122,049 participants of European ancestry, we identified 2446 independent SNPs in 906 genome-wide significant loci, of which 364 loci were novel (Supplementary Fig. 3A, Supplementary Data 1). Using LDSC and GWAS summary statistics, we estimated the inflation factor (λ) and heritability (h^2^) of BMI. We observed λ of 1.599, which was typical in GWAS with very large sample sizes, and h^2^ of 0.188 (SE of 0.008) of BMI. The LDSC intercept of 1.053 (SE 0.011) and small ratio of 0.062 (SE of 0.013), indicate the majority of the inflation was due to polygenicity of BMI, not confounding. For AA participants, we identified 18 significant loci in the MVP. In the meta-analysis combining the MVP and the AAAGC, we discovered a total of 100 independent SNPs in 41 BMI-associated loci, of which 33 loci were novel for African ancestry and 6 loci were novel compared to previous BMI GWAS in any ancestry group (Supplementary Fig. 3B, Supplementary Data 2).

Using the total of 2,446 and 100 independent SNPs (lead SNPs and secondary SNPs: pair-wise LD with lead SNPs *r*^2^ < 0.1 and *p* < 5 × 10^−8^) in EA and AA participants, respectively, we conducted a weighted GRS_BMI_ analysis for EA and AA participants using the meta-analysis beta coefficients, after removing MVP, as weights for the GRS. Several statistically significant SNPs from the main GWAS meta-analysis were only available in MVP, therefore the final numbers of SNPs in the GRS were 2428 and 94 in EA and AA, respectively. The EA-specific GRS_BMI_ was associated with BMI (*p* < 10^−314^) and explained 5.4% of BMI variance in the MVP EA participants. The AA-specific GRS_BMI_ was also associated with BMI (*p* < 10^−314^) and explained 0.9% of BMI variance in the MVP AA participants. Using SNPs and beta-coefficients identified in the EA-specific BMI GWAS, the weighted EA GRS_BMI_ was associated with BMI but explained only 1.5% of BMI variance in 55,525 AA participants.

### Nongenetic BMI associations with outcomes

We tested associations of BMI with 1,244 disease codes, for which there were ≥200 cases and controls in EA participants, drawn from phenotype codes defined in previous PheWAS analyses^18^. In this cross-sectional analysis, 661 phecodes were associated with BMI after correction for multiple testing (see Supplementary Data 3). 75% (*n* = 493) of these phecodes were positively associated with BMI (increased risk with higher BMI). Expected findings included positive associations with obesity/overweight and T2DM, and negative associations with anorexia.

### Phenome-wide Mendelian randomization of BMI and outcomes

**Fig. 1:**
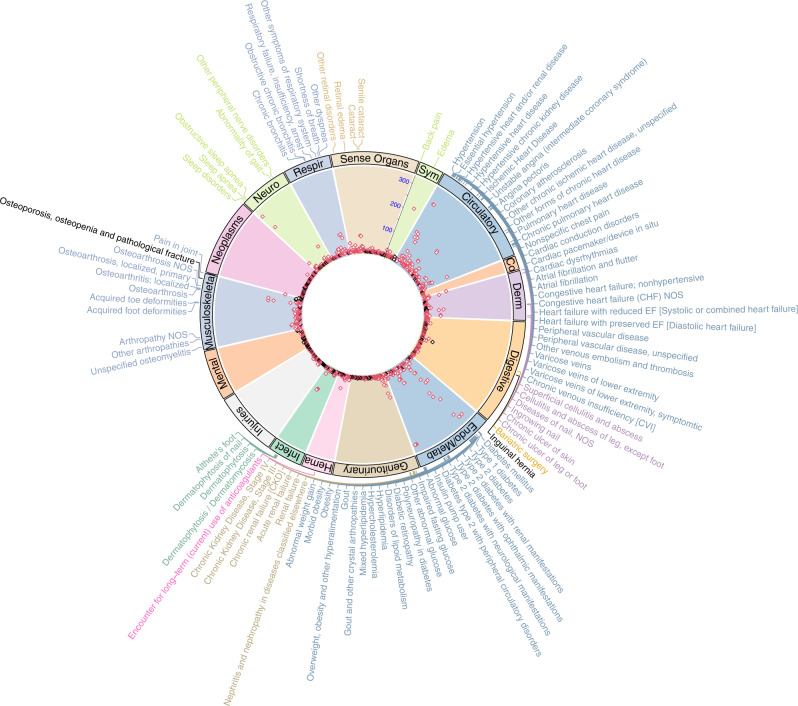
Summary of phenome-wide Mendelian randomization analysis using GRS_BMI_. Red dots represent a statistically significant positive association with GRS_BMI_ ( ↑ GRS_BMI_ = ↑ disease risk), and black dots represent a statistically significant negative association in the MR analysis after multiple testing correction (two-sided test with nominal *p*-value less than 4.02 × 10^−5^). Top 100 associations with lowest p-values in the MR analysis are labeled including 98 positive associations (colored font) and two negative associations (black font) from 13 systems. Abbreviations (clockwise): Circulatory: circulatory system; Co: congenital anomalies; Derm: dermatologic; Endo/Metab: endocrine/metabolic; Hema: hematopoietic; Infect: infectious diseases; Injuries: injuries & poisonings; Mental: mental disorders; Neuro: neurological; Respir: respiratory; Sym: symptoms.

We conducted MR analysis of the 1244 phecodes in up to 174,915 EA participants. Of the phecodes associated with standardized BMI, MR results were also associated (threshold *p* < 4.02 × 10^−5^) with 316 codes across 16 disease groups (Table 2, Fig. 1, Supplementary Data 4). The presence and magnitude of a number of associations, including T2DM (OR = 2.64 per SD of BMI [95% CI 2.54–2.75]), sleep apnea (OR = 2.36 [95% CI 2.26–2.46]), hypertension (OR = 2.20 [95% CI 2.10-2.30]), ischemic heart disease (OR = 1.67 [95% CI 1.60–1.74]), and asthma (OR = 1.25 [95% CI 1.17–1.33]), were consistent with recent MR studies of BMI for these diseases^10,11,19^. After accounting for multiple testing, there were a number of MR associations with circulatory system and metabolic diagnoses that were not previously reported to be significant. These included a broad set of cardiovascular diagnoses that confer increased mortality, such as the subtypes of heart failure with preserved ejection fraction and heart failure with reduced ejection fraction, heart block, and cardiomyopathy. In addition, we identified associations with many other major conditions that increase morbidity and mortality, including major respiratory, genitourinary, digestive, musculoskeletal and sensory conditions such as respiratory failure, cholelithiasis, chronic renal failure, diabetic retinopathy and macular degeneration (Table 2). In a sensitivity analysis, these associations in the MR analyses were essentially the same in analyses using an alternative GRS_BMI_ based on beta coefficients derived from the meta-analysis that included MVP (correlation coefficient r = 0.999, Supplementary Figs. 4–6).

**Table 2:** Selected phenome-wide associations between GRS_BMI_ and clinical diseases in the MR analysis of non-Hispanic EAs

Phecode	Description	Phenotype group	GRS_BMI_-MR associations				
			BETA	SE	OR	95% CI	*P*-value
POSITIVE CONTROLS							
278.11	Morbid obesity	Endocrine/metabolic	2.35	0.04	10.44	(9.76, 11.18)	<10^−314^
260.6	Anorexia	Endocrine/metabolic	−1.20	0.20	0.30	(0.20, 0.44)	1.09 × 10^−09^
INCREASED RISK ASSOCIATED WITH BMI							
401	Hypertension	Circulatory system	0.79	0.02	2.20	(2.10,2.30)	2.06 × 10^−263^
428.1	Congestive heart failure (CHF) NOS	Circulatory system	0.82	0.03	2.26	(2.11, 2.41)	6.62 × 10^−129^
428.4	Heart failure with preserved EF	Circulatory system	1.26	0.10	3.53	(2.90, 4.28)	3.69 × 10^−37^
428.3	Heart failure with reduced EF	Circulatory system	0.87	0.09	2.39	(2.02, 2.84)	1.10 × 10^−23^
411	Ischemic Heart Disease	Circulatory system	0.51	0.02	1.67	(1.60, 1.74)	8.29 × 10^−135^
440.2	Atherosclerosis of the extremities	Circulatory system	0.48	0.06	1.61	(1.44, 1.80)	4.82 × 10^−17^
454.11	Varicose veins of lower extremity, symptomatic	Circulatory system	1.03	0.07	2.80	(2.45, 3.20)	2.99 × 10^−51^
427.2	Atrial fibrillation and flutter	Circulatory system	0.48	0.03	1.61	(1.52, 1.71)	6.26 × 10^−55^
426.2	Atrioventricular [AV] block	Circulatory system	0.53	0.06	1.70	(1.51, 1.92)	6.86 × 10^−18^
452.2	Deep vein thrombosis [DVT]	Circulatory system	0.51	0.07	1.66	(1.45, 1.90)	8.92 × 10^−14^
415	Pulmonary heart disease	Circulatory system	0.55	0.05	1.72	(1.55, 1.92)	3.10 × 10^−24^
681	Superficial cellulitis and abscess	Dermatologic	0.53	0.03	1.70	(1.62, 1.79)	5.82 × 10^−91^
707.2	Chronic ulcer of leg or foot	Dermatologic	0.82	0.05	2.27	(2.05, 2.51)	6.39 × 10^−57^
696.4	Psoriasis	Dermatologic	0.25	0.05	1.28	(1.16, 1.41)	5.11 × 10^−7^
550.5	Ventral hernia	Digestive	0.63	0.07	1.88	(1.64, 2.15)	2.92 × 10^−20^
525.1	Loss of teeth or edentulism	Digestive	0.27	0.03	1.31	(1.23, 1.39)	1.42 × 10^−19^
574	Cholelithiasis and cholecystitis	Digestive	0.32	0.05	1.38	(1.26, 1.51)	1.75 × 10^−12^
244	Hypothyroidism	Endocrine/metabolic	0.15	0.03	1.16	(1.10, 1.22)	1.43 × 10^−07^
250.2	Type 2 diabetes	Endocrine/metabolic	0.97	0.02	2.64	(2.54,2.75)	<10^−314^
585.3	Chronic renal failure [CKD]	Genitourinary	0.60	0.03	1.82	(1.71, 1.94)	1.78 × 10^−76^
585.1	Acute renal failure	Genitourinary	0.64	0.05	1.90	(1.74, 2.08)	1.32 × 10^−44^
591	Urinary tract infection	Genitourinary	0.23	0.04	1.25	(1.17, 1.34)	8.48 × 10^−11^
594	Urinary calculus	Genitourinary	0.17	0.03	1.19	(1.11, 1.27)	5.13 × 10^−07^
286	Coagulation defects	Hematopoietic	0.36	0.06	1.44	(1.28, 1.61)	3.32 × 10^−10^
110	Dermatophytosis / Dermatomycosis	Infectious diseases	0.48	0.02	1.61	(1.54, 1.69)	4.13 × 10^−92^
41	Bacterial infection NOS	Infectious diseases	0.39	0.05	1.48	(1.35, 1.62)	3.83 × 10^−17^
41.1	Staphylococcus infections	Infectious diseases	0.55	0.08	1.74	(1.50, 2.01)	1.38 × 10^−13^
38	Septicemia	Infectious diseases	0.43	0.07	1.54	(1.34, 1.77)	1.18 × 10^−09^
875	Non-healing surgical wound	Injuries/poisonings	0.68	0.10	1.97	(1.64, 2.38)	8.02 × 10^−13^
871.4	Open wound of toe(s)	Injuries/poisonings	0.97	0.22	2.62	(1.72, 4.00)	6.88 × 10^−06^
871.3	Open wound of foot except toe(s) alone	Injuries/poisonings	0.77	0.15	2.15	(1.61, 2.86)	1.64 × 10^−07^
740.9	Osteoarthrosis NOS	Musculoskeletal	0.48	0.02	1.61	(1.55,1.67)	4.15 × 10^−128^
716.9	Arthropathy NOS	Musculoskeletal	0.37	0.03	1.45	(1.36, 1.54)	7.02 × 10^−33^
710.1	Osteomyelitis	Musculoskeletal	0.66	0.07	1.93	(1.67, 2.23)	4.96 × 10^−19^
720.1	Spinal stenosis of lumbar region	Musculoskeletal	0.38	0.04	1.46	(1.33, 1.59)	2.96 × 10^−17^
735.2	Acquired toe deformities	Musculoskeletal	0.36	0.04	1.44	(1.34, 1.55)	1.63 × 10^−22^
327.3	Sleep apnea	Neurological	0.86	0.02	2.36	(2.26, 2.46)	<10^−314^
327.71	Restless legs syndrome	Neurological	0.45	0.06	1.57	(1.40, 1.76)	1.80 × 10^−14^
509.1	Respiratory failure	Respiratory	0.62	0.08	1.85	(1.59, 2.16)	6.40 × 10^−15^
495	Asthma	Respiratory	0.22	0.03	1.25	(1.17, 1.33)	1.05 × 10^−10^
366	Cataract	Sense organs	0.34	0.02	1.41	(1.36, 1.47)	7.23 × 10^−62^
362.2	Degeneration of macula & posterior retina	Sense organs	0.29	0.03	1.33	(1.25, 1.43)	1.99 × 10^−17^
365	Glaucoma	Sense organs	0.18	0.03	1.19	(1.13, 1.25)	2.15 × 10^−11^
782.3	Edema	Symptoms	0.94	0.03	2.55	(2.41, 2.71)	4.89 × 10^−212^
DECREASED RISK ASSOCIATED WITH BMI							
550.1	Inguinal hernia	Digestive	−0.55	0.04	0.58	(0.54, 0.63)	1.11 × 10^−45^
54	Herpes simplex	Infectious diseases	−0.37	0.07	0.69	(0.61, 0.79)	4.77 × 10^−08^
317.1	Alcoholism	Mental disorders	−0.22	0.03	0.80	(0.75, 0.85)	2.61 × 10^−14^
317	Alcohol-related disorders	Mental disorders	−0.22	0.03	0.81	(0.77, 0.85)	3.69 × 10^−17^
743.1	Osteoporosis	Musculoskeletal	−0.45	0.05	0.64	(0.58, 0.70)	1.43 × 10^−20^

Alpha level is p-value of 4.02 × 10^−5^ (Bonferroni correction: 0.05 /1244). PheWAS disease groups were defined by the PheWAS method^18^ OR (odds ratio) was calculated by per SD increase of BMI. A complete list of all statistically significant MR associations is shown in Supplementary Table 4.

To further test for the validity of MR associations with the phecodes significantly associated by initial MR PheWAS analyses, we conducted a set of additional MR association tests, including MR inverse-variance weighted (IVW), median-weighted and Egger regression analyses (Supplementary Data 5). Associations remained significant for most of the phenotypes in one or more of the association tests. All but one phecodes had *p*-value less than 0.05 in the IVW MR analysis, and 78% phecodes had *p* value less than 0.05 in the MR Egger analysis, which addresses pleiotropy but is known to be under-powered.

The burden of the 316 disease codes associated with genetically influenced BMI in EA increased across increasing BMI categories (Supplementary Data 6). The number of disease codes was higher in obese and severely obese persons (for BMI ≥ 30, mean 26.7 [median 23], for BMI ≥ 40, mean 32.1 [29]) compared with BMI < 25 (mean 20.2[17]). There was a trend (*p* < 0.001) for an increasing number of associated disease codes across BMI categories (Supplementary Data 6). Among individuals with BMI ≥ 40, 69.9% had more than 20 codes associated with genetically influenced BMI, compared with 40.4% in those with BMI < 25 (Supplementary Data 6).

The MR analysis also showed a negative (protective) direction of association with BMI for 3.5% (*n* = 11) phecodes (i.e., decreased risk with higher BMI), including inguinal hernia, osteoporosis, and alcoholism. An additional 197 phecodes from 16 disease systems were associated with BMI but were not associated (*p* > 0.05) with genetically influenced BMI (Supplementary Data 7).

For participants of African ancestry, we examined the association of the GRS_BMI_ based on the 2428 genome-wide associated SNPs in the EA meta-analysis to increase strength of the instrumental variable. Of the phecodes associated with standardized BMI, MR association results were also statistically significant (*p* < 6.00 × 10^−5^) for 61 codes across 11 disease groups using weights from the EA meta-analysis excluding MVP (Supplementary Data 8). These included T2DM and several major diabetic complications, sleep apnea, hypertension, congestive heart failure and heart failure with preserved ejection fraction, ischemic heart disease, and chronic kidney disease. There were 34 phecodes associated using the 2428 SNP GRS_BMI_ with weights from the AAAGC (Supplementary Data 8). Of these 34, all were associated with EA GRS with EA weights. We also constructed a GRS_BMI_ based on 94 independent SNPs identified by the largest BMI meta-analysis of AA participants combining data from MVP and the AAAGC but weighted using the effect sizes from AAAGC alone^20^. Four phecodes were associated in a positive direction with genetically influenced BMI in AA, after controlling for multiple testing (Supplementary Data 8). Apart from BMI-related phecodes (overweight, obesity, morbid obesity), we also noted a relationship between genetically influenced BMI and sleep apnea (OR = 2.09, [95% CI: 1.48–2.96], *p* = 3.04 × 10^−5^).

Finally, to explore for the potential of reverse causality in the associations between BMI and conditions in our PheWAS, we selected 10 traits across a range of disease for which GWAS summary data are available (see Results for PheWAS and Network analysis in the Supplementary Methods). As expected from prior research, there is a strong bidirectional (two-way) “causal” effect between body mass index and type 2 diabetes. However, there was no evidence of significant (inverse variance weighted-based MR *P*-value < 0.005) bidirectional effect to BMI for any of the other nine traits (see Supplementary Table 1 in the Supplementary Methods).

### Analysis of disease comorbidity network

**Fig. 2:**
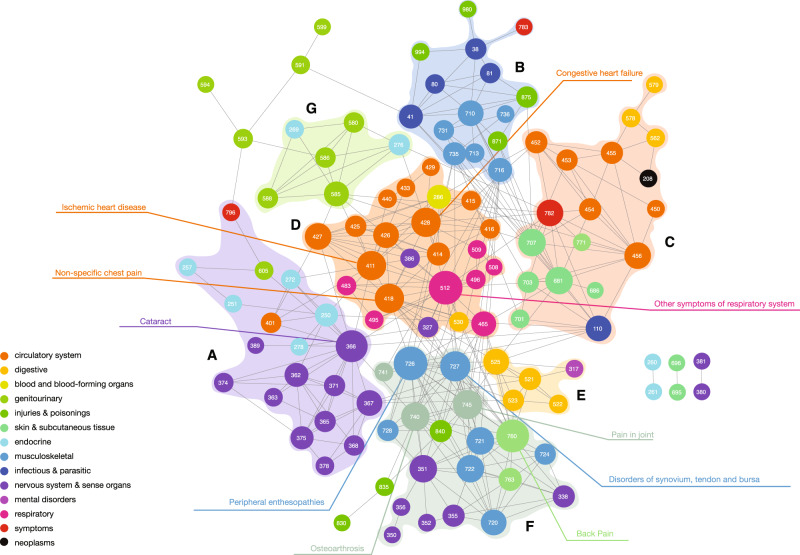
Phenotypic network map. Nodes represent phecodes at the three-digit level and the links represent significant disease-disease associations (*ϕ*-correlation). Node size is proportional to network connectivity (degree). Individual disease communities (A-G) are described in the Supplementary Materials.

We created a human disease network of phecode-based diseases and disorders to evaluate the comorbidity patterns among 134 3-digit codes constructed from 316 phecodes associated with genetically influenced BMI from the phenome-wide MR findings. We included only conditions that are strongly associated with genetically defined BMI to focus on networks of conditions that are grounded in strong evidence for association consistent with a “causal” association with BMI and less likely to be confounded by other factors. The resulting network map (117 higher level phecodes with 360 links) identified many diseases associated with BMI in MR analyses that co-occur in several distinct patterns that might indicate the typical clinical burden for individual persons (Fig. 2). The highly connected nodes (i.e., hubs) represent diagnoses that tend to co-occur with many others (Supplementary Data 9), reflecting common patterns of symptoms or of specific diseases. The most connected diseases included disorders of attachment of ligaments or tendons to bone (“peripheral enthesopathies”) and respiratory symptoms, each strongly correlated with 20 disease codes. In a subset of phecodes with multiple connections in the network analysis (degree > 5 in Supplementary Data 9), evidence for MR association in one or more tests was strong and consistent with a similar magnitude and direction of effect (Supplementary Data 5). Among the common disease diagnoses associated with 10 or more conditions were conditions such as cardiovascular diseases, acute upper respiratory illness, and renal failure that account for leading causes of death in the United States^21^.

We identified seven disease communities (see Supplementary Fig. 7A-G)—groups of diseases associated with genetically influenced BMI that tend to co-occur in the larger disease comorbidity network—using permutation-based statistical tests (*p* value < 0.0041, 0.05/12 communities). These communities were comprised of diseases from multiple disease systems (e.g., Community A: circulatory, endocrine, nervous systems, genitourinary, and general symptoms). Underscoring the extensive interconnectedness of the disease codes associated with genetically influenced BMI in this network, each of the top ten nodes was connected with over a dozen disease codes (ranging from 13 to 20) within a community and also with disease codes in up to six different communities (Supplementary Data 9).

## Discussion

We discovered a total of 370 novel genetic loci for BMI in samples of European (364) and African descent (6), through a large-scale ancestry-specific meta-analysis of European and African ancestry GWAS, including the MVP. We incorporate these newly discovered genetic variants into the largest phenome-wide MR analysis performed to date, identifying several hundred diseases from across 16 different disease categories in AA and EA US Veterans for which BMI is implicated as a genetically associated risk factor. While we confirmed a number of previously reported associations, including those recently reported in two recent MR analyses of the UK Biobank^13,19^, we also identified many conditions not associated with genetically influenced BMI in previous MR analyses. There was a striking increase in the burden of MR-associated disease codes across increasing categories of BMI. In our analysis of disease comorbidity networks that incorporates all strongly associated conditions from our phenome-wide MR analysis, there were seven communities of diseases with extensive intra- and inter-community connections, underscoring new insights into the complex genetic underpinnings of obesity and its impact on diseases in the population.

The high prevalence of obesity has continued to grow worldwide and across the United States^1,22,23^, including users of the Veterans Administration health care system^24,25^. For example, from 2007–2008 to 2015–2016, the age-standardized prevalence of obesity increased in US adults from 33.7% to 39.6%, respectively^23^. In the Global Burden of Disease (GBD) analysis, high BMI accounts for 4 million excess deaths per year, the majority being related to cardiovascular disease^1^. In the same study, 20 disease endpoints were identified through conventional epidemiological studies but no MR analysis was cited to support causality. The urgent need to understand these links is underscored by evidence from the recent pandemic of SARS-CoV-2 for an increased risk of mortality in obese COVID-19 patients^26^ as well as increased risk for severe COVID-19 illness associated with obesity supported by two-sample MR studies^27–29^.

Through our MR analysis, we confirm and extend the findings for strong associations with common conditions in the GBD analysis in EA and AA US Veterans. Furthermore, we also provide novel strong evidence for associations of BMI with risk for many specific major circulatory system diseases, including heart failure with preserved ejection fraction, atrial fibrillation, aortic valve disease, venous thromboembolism, and abdominal aortic aneurysm. These strong associations highlight a broader impact of excess adiposity on cardiovascular disease morbidity than previously appreciated. Additionally, we observed associations in the MR analysis with a range of other conditions that are either life-threatening or adversely affect quality of life. These included life-threatening conditions of the genitourinary (chronic renal failure) and respiratory (respiratory failure, asthma) systems as well as conditions associated with substantial morbidity in the musculoskeletal and dermatologic systems. We noted a limited number of inverse associations that are consistent with prior observational studies, such as inguinal hernia^30^ and osteoporosis^31^. We also note other inverse disease associations in the MR analysis that warrant further investigation, such as protective associations with selected viral infectious diseases (herpes simplex virus) and substance abuse. Given the lack of published studies in populations of African descent, nearly all our MR association findings are novel in that population.

Our analysis of disease comorbidity networks incorporated over 300 conditions and identified seven communities with extensive intra- and inter-community associations of multiple BMI-associated conditions. While several communities were enriched for cardiovascular diseases, others were enriched for skin diseases, renal diseases, pulmonary diseases, and disorders of the eye and other sensory organs. This extensive set of conditions with evidence of association with genetically influenced BMI extends prior evidence for the clinical co-occurrence of multiple comorbidities conferred by obesity. Our findings are also consistent with a recent study showing substantial benefit on multiple outcomes, in obese diabetic patients treated with metabolic weight reduction surgery^32^.

In summary, we harnessed genetic variation discovered in large-scale meta-analysis of both European and African ancestry GWAS, revealing associations of increased genetic risk of BMI for several hundred diseases in a phenome-wide Mendelian randomization analysis and intra- and inter-community connections in an extensive disease comorbidity network. Our findings underscore the broad impact of obesity on multiple interconnected chronic and acute diseases and highlight the public health imperative to prevent and treat obesity in order to reduce downstream morbidity and mortality from numerous obesity-associated diseases.

## Methods

### Study participants

The design of the MVP has been previously described^33^. Briefly, individuals aged 19 to 104 years with the mean age of 62 years have been recruited from over 60 Veterans Health Administration medical centers nationwide since 2011. Each veteran’s EHR is being integrated into the MVP biorepository, including inpatient International Classification of Diseases (ICD9/10) diagnosis codes, Current Procedural Terminology (CPT) procedure codes, clinical laboratory measurements, and reports of diagnostic imaging modalities.The MVP research database integrates the extensive EHR data from each enrolled Veteran. MVP has received ethical and study protocol approval by the VA Central Institutional Review Board in accordance with the principles outlined in the Declaration of Helsinki.

### Phenotype

EHR data from clinical examinations were available for MVP participants from as early as 2003. BMI is calculated as the weight (in kilograms) divided by the height (in meters) squared. We calculated the average BMI using all measurements within a three-year window around the date of MVP enrollment (i.e., 1.5 years before/after the date of enrollment), excluding height measurements that were >3 inches (0.0762 meters) or weight measurements >60 pounds (27.22 kilograms) from the average of each participant^25^.

### Genetic data and genome-wide association analysis

DNA extracted from participants’ blood was genotyped using a customized Affymetrix Axiom® biobank array, the MVP 1.0 Genotyping Array, with genotype imputation to the 1000 Genomes reference panel^34^ as detailed in the Supplementary Materials. We used both self-reported ethnicity and genetic ancestry to define non-Hispanic European American (EA) and Non-Hispanic African American (AA) participants in the MVP.

Genetic association with BMI in the MVP cohort was examined separately among non-Hispanic EA (*N* = 215,734) and non-Hispanic AA (N = 55,525) participants. For each ancestry group, BMI was stratified by sex and adjusted for age, age-squared, and the top ten genotype-derived principal components of ancestry in a linear regression model. The resulting residuals were transformed to approximate normality using inverse normal scores. Imputed and directly measured genetic variants were tested for association with the inverse normal transformed residuals of BMI through linear regression assuming an additive genetic model.

We performed ancestry-specific inverse-variance weighted fixed-effects meta-analysis using METAL^35^. For EA participants, we meta-analyzed GWAS results from MVP, UK Biobank, and the Genomic Investigation of Anthropometric Traits (GIANT) Consortium. For AA participants, we meta-analyzed GWAS results from MVP with the African Ancestry Anthropometry Genetics Consortium (AAAGC) consortium. GWAS results were summarized using FUMA (http://fuma.ctglab.nl/)^36^. Novel loci were defined as those with genome-wide significance (p < 5 × 10^−8^) and a distance > 500 kb from previously published variants^14,20,37,38^.

### Phenome-wide association and Mendelian randomization analyses

We constructed EA- and AA-specific weighted genetic risk scores of BMI (GRS_BMI_) to perform phenome-wide MR analysis, using the independent and genome-wide significant SNPs from the respective BMI meta-analysis. Beta coefficients from the respective ancestry-specific meta-analyses, excluding MVP, were used as weights for each GRS in order to avoid overfitting.

The phenotypes characterizing disease diagnoses include the full catalog of phecodes (*N* = 1813) from sixteen disease systems determined at participant enrollment^16^, and all analyses were limited to phecodes with at least 200 cases and 200 controls in the 174,531 EA or 49,695 AA participants (*N* = 1244 for EA and *N* = 833 for AA).

For the PheWAS, logistic regression models were used to assess the association between standardized BMI [(bmi-mean(bmi))/sd(bmi)] and phecodes, adjusted for age, sex and the top 10 genotype-derived principal components. Phenotypes were considered associated if they had a *p*-value less than 4.02 × 10^−5^ and 6.00 × 10^−5^ (Bonferroni corrected *p*-value of 1,244 and 833 traits) for EA and AA, respectively.

To search for evidence of genetic association of BMI with BMI-associated traits, we then conducted the phenome-wide MR analysis in EA and AA participants separately using ancestry-specific GRS_BMI_ as the genetic instrumental variable^6^. We used the same set of phecodes included in the PheWAS. To explore in depth the validity of MR associations, we examined further for associations using inverse-variance weighted (IVW), MR Egger regression and weighted median MR analysis^39^ for all PheWAS phecodes that were significant in the phenome-wide PRS analysis. To identify significant phenotype associations, we used the same Bonferroni correction for the PheWAS to account for the total number of phenotypes (*N* = 1244 for EA and *N* = 833 for AA) tested in the initial MR PheWAS. Details of phenomic data quality control, case definitions, and association analysis are described in the Supplementary Materials.

### Disease comorbidity networks

We created a phenotypic network map to evaluate the comorbidity patterns among the diagnosis codes for which there was plausible evidence (*p* < 4.02 × 10^−5^) for BMI in the causal pathway in the MR analysis. The nodes in the network represent disease diagnosis codes rounded up to their correspondent 3-digit level and links between nodes represent the strength and significance of disease co-occurrence (comorbidity). To measure the comorbidity strength for diseases *i* and *j*, we used the *ϕ*-correlation *ϕ*_*ij*_^40^, and determined their significance using *t*-test corrected for multiple testing. The *ϕ*-correlation, which is Pearson’s correlation for binary variables, can be expressed mathematically as the following equation (Eq. 1):

1 $${\phi}_{{ij}}=\frac{{C}_{{ij}}N-{P}_{i}{P}_{j}}{\sqrt{{P}_{i}{P}_{j}(N-{P}_{i})(N-{P}_{j})}}$$

where *C*_*ij*_ is the number of patients affected by both diseases, *N* is the total number of patients in the population and *P*_*i*_ and *P*_*j*_ are the prevalence of diseases *i* and *j*. Only pairs with adjusted p-value < 0.05 and *ϕ* >= 0.2 were considered. Next, we used the network structure to identify groups of diseases (i.e., communities) that show higher comorbidity (links in the network) among diseases in the group in comparison with the remaining diseases in the network. We applied the Louvain community detection algorithm^41^ and the qstest method^42^ for evaluation of community significance.

### Reporting summary

Further information on research design is available in the Nature Portfolio Reporting Summary linked to this article.

## Supplementary Information

Supplementary Information

Description of Additional Supplementary Files

Supplementary Data 1-9

Reporting Summary

## Data Availability

Due to US Department of Veterans Affairs (VA) regulations and our ethics agreements, the individual-level data sets used for this study are not permitted to leave the Million Veteran Program (MVP) research environment and VA firewall. This limitation is consistent with other MVP studies based on VA data. However, the MVP data are made available to researchers with an approved VA and MVP study protocol. The full summary level association results from genome-wide association analyses in the MVP and the meta-analysis from this report are available through a standard application to dbGaP (accession number phs001672). The only restriction is that use of the data is limited to health/medical/biomedical purposes and does not include the study of population origins or ancestry. Use of the data does include methods development research (e.g., development and testing of software or algorithms) and requestors agree to make the results of studies using the data available to the larger scientific community. Summary statistics of BMI GWAS from the GIANT and UK Biobank meta-analysis are available from: https://portals.broadinstitute.org/collaboration/giant/index.php/GIANT_consortium_data_files. Summary association statistics from AAAGC BMI meta-analyses are available from dbGaP at accession number phs000930.
