# Supplementary material for: Genomics and phenomics of body mass index reveals a complex disease network
Source: Nat Commun. 2022 Dec 29;13:7973. doi: 10.1038/s41467-022-35553-2 (PMC9798356; doi:10.1038/s41467-022-35553-2)
Supplement: Supplementary file 1 — Supplementary Information [file 41467_2022_35553_MOESM1_ESM.pdf]

Supplementary Materials

Supplementary Figures Pages 2-8

Supplementary Notes Pages 9-13

Million Veteran Program (MVP) Acknowledgement Pages 14-19

References Page 20

Supplementary Figure 1. Principal component plot for multi-ancestry participants in the Million Veteran Program.

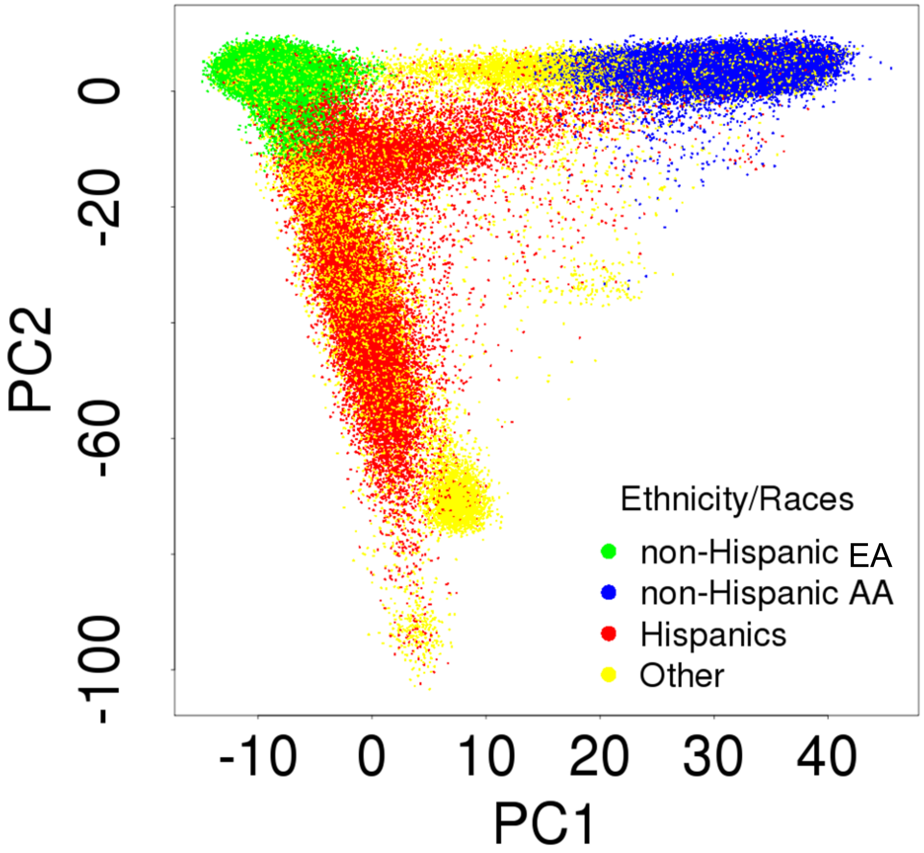

Supplementary Figure 1 - A principal component analysis was performed on all MVP samples. The first two PCs were plotted for diverse MVP genetic ancestry including non-Hispanic EA (i.e., non-Hispanic European ancestry), non-Hispanic AA (i.e., non-Hispanic African ancestry), Hispanics and all others.

Supplementary Figure 2. Overview of the study design and analysis workflow

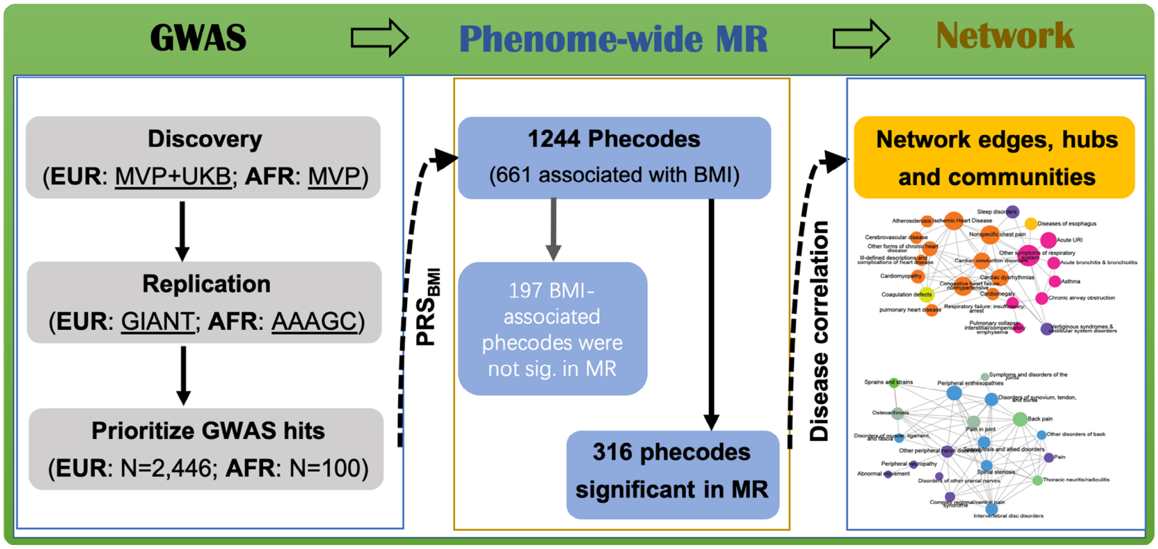

Supplementary Figure 3. Manhattan plots for BMI GWAS in European ancestry in the meta-analysis of MVP+GIANT+UKB (A) and African ancestry in the meta-analysis of MVP+AAAGC (B)

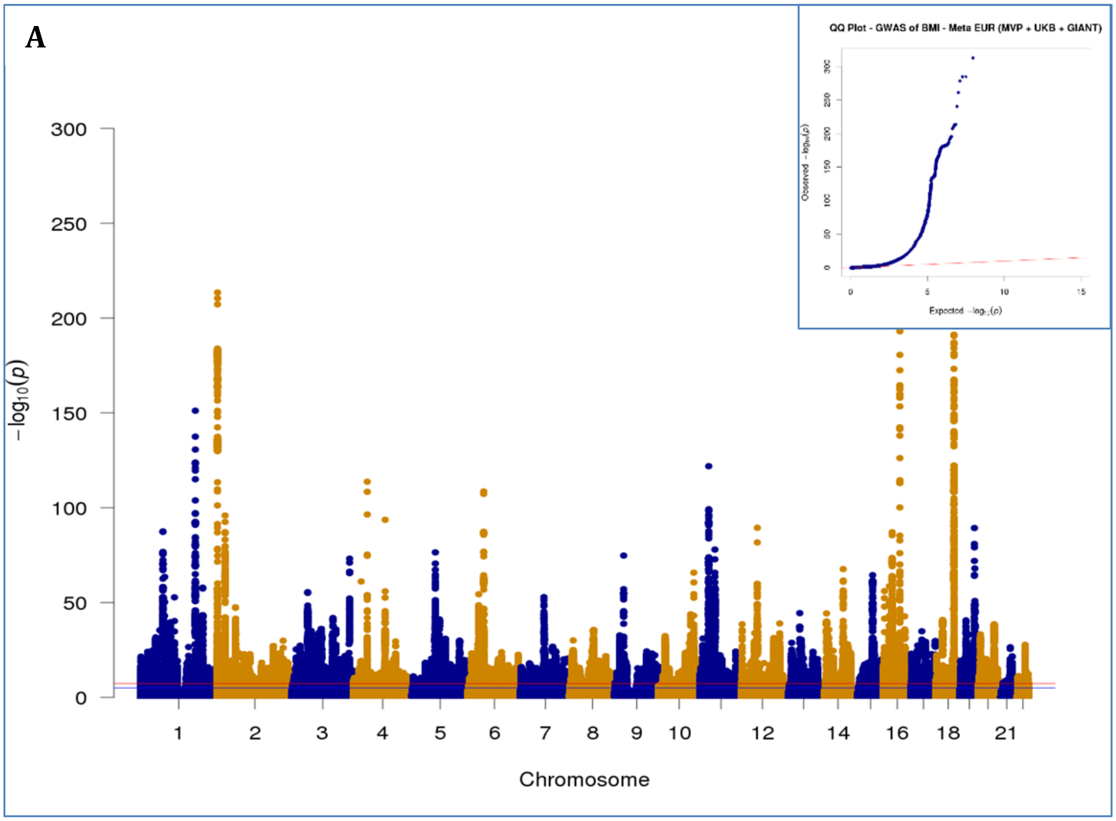

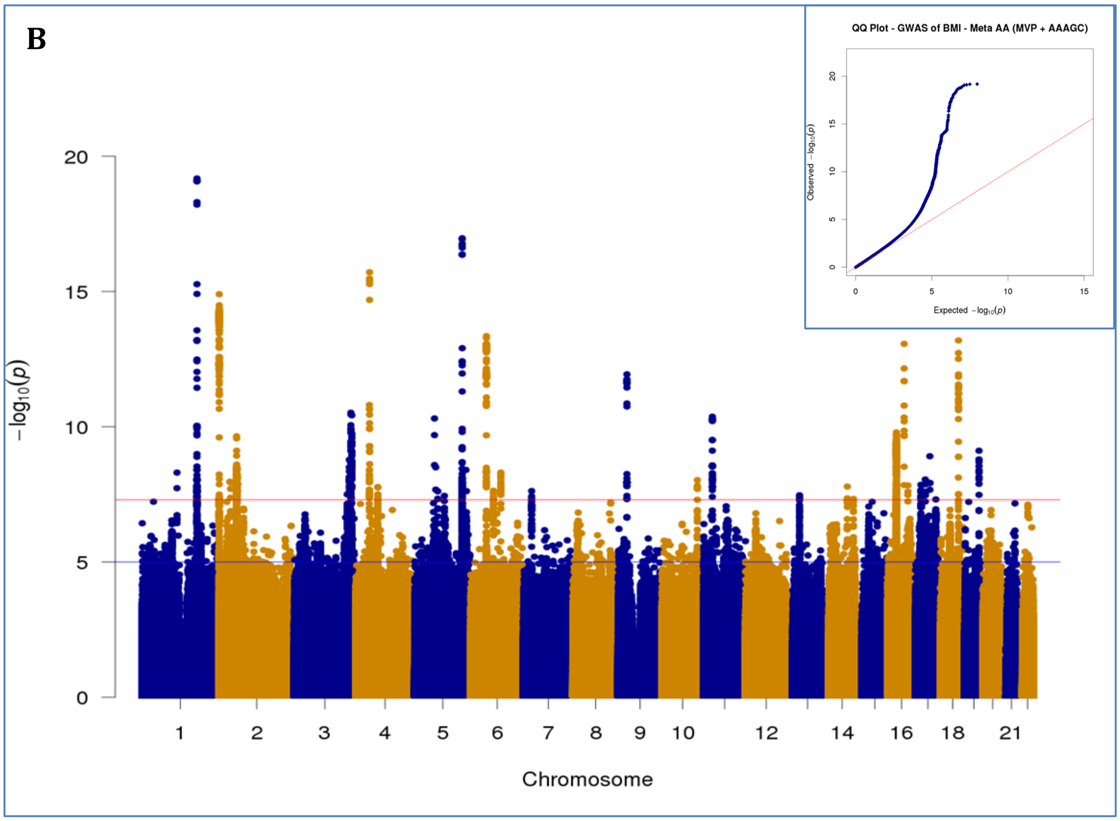

Supplementary Figure 4. Comparison of BMI effect estimates and effect allele frequencies of 941 genome-wide significant lead SNPs between samples of European ancestry in the meta-analysis of MVP+GIANT+UKB.

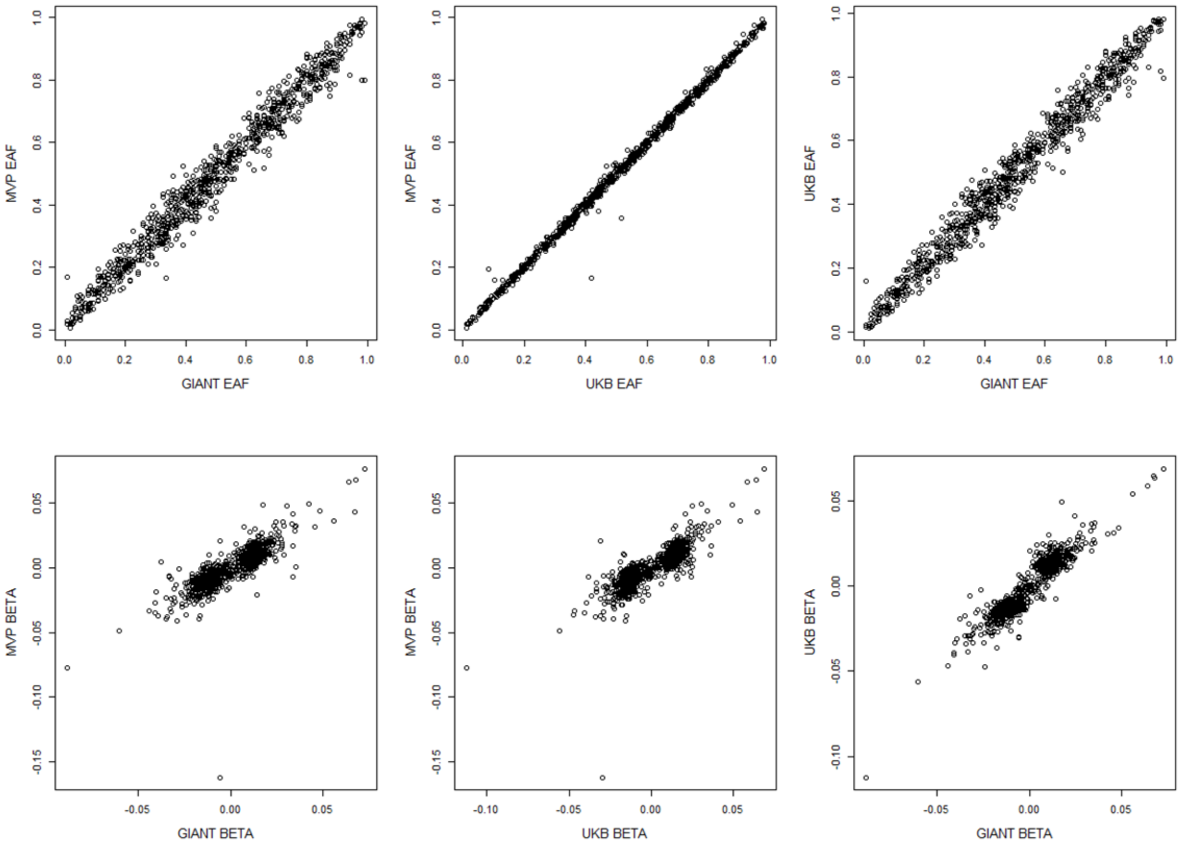

Supplementary Figure 5. Comparison of BMI effect estimates and effect allele frequencies of genome-wide significant SNPs between samples of European ancestry and African ancestry in the MVP.

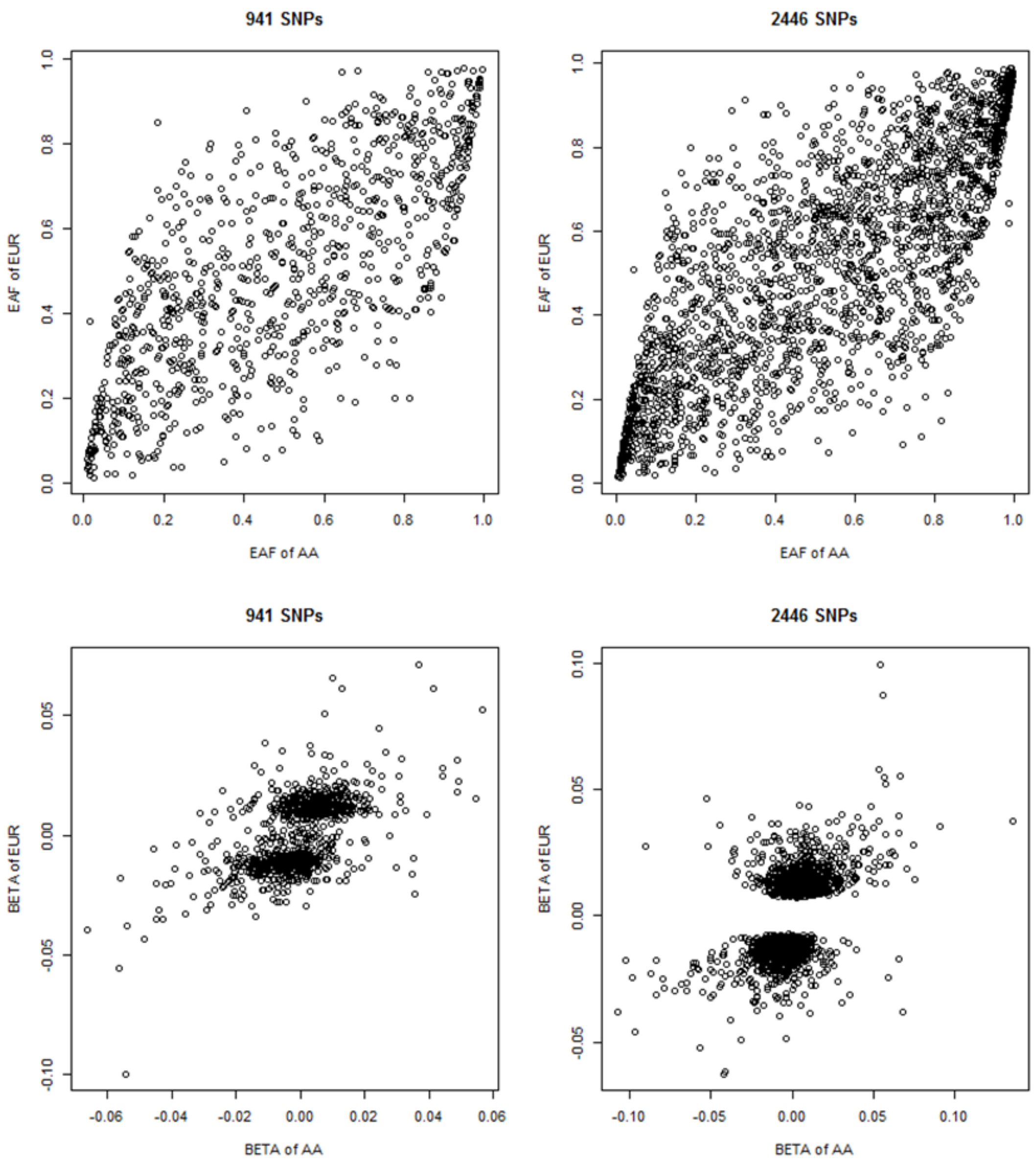

Supplementary Figure 6. Comparison of two weighted GRS_{BMI} based on genetic effect estimates from the European ancestry meta-analyses of MVP+UKB+GIANT versus UKB+GIANT. The ORs of 316 significant associations from the phenome-wide MR analyses were calculated separately and compared using Pearson correlation test.

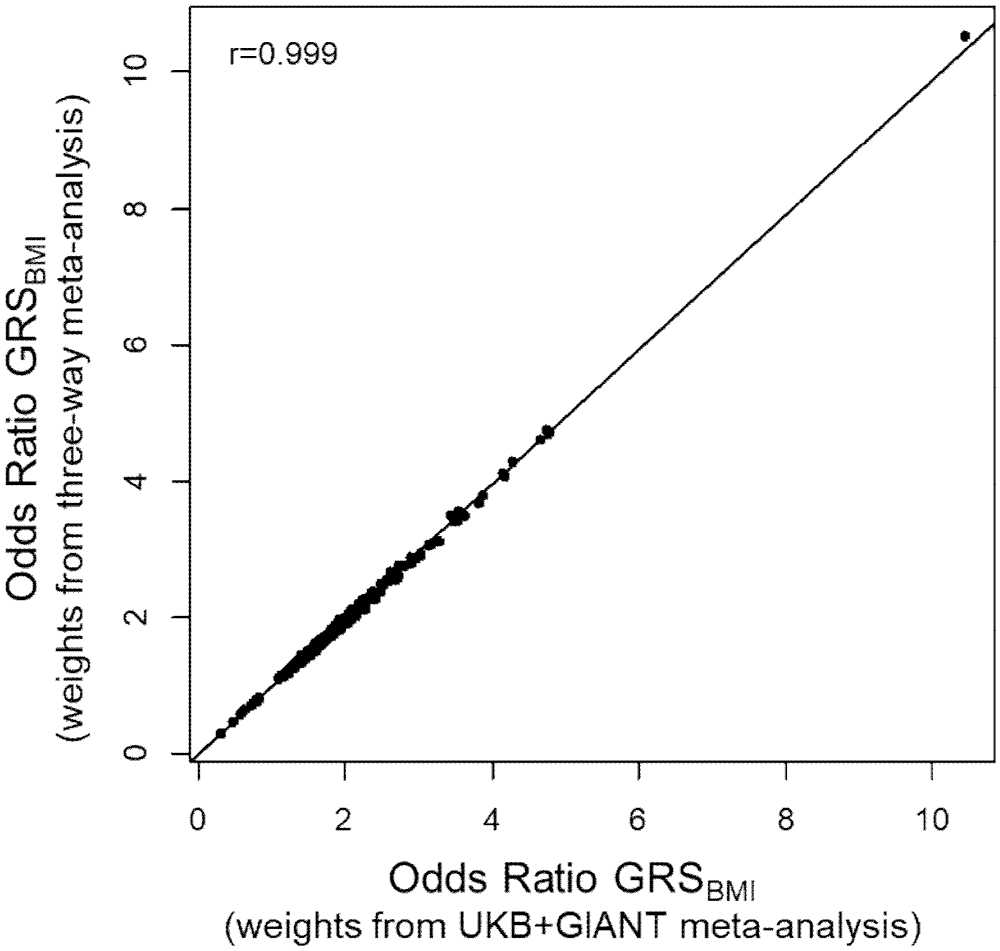

Supplementary Figure 7. Disease communities (A-G) identified by the network analysis of BMI-driving disease diagnosis codes from the phenome-wide MR study.

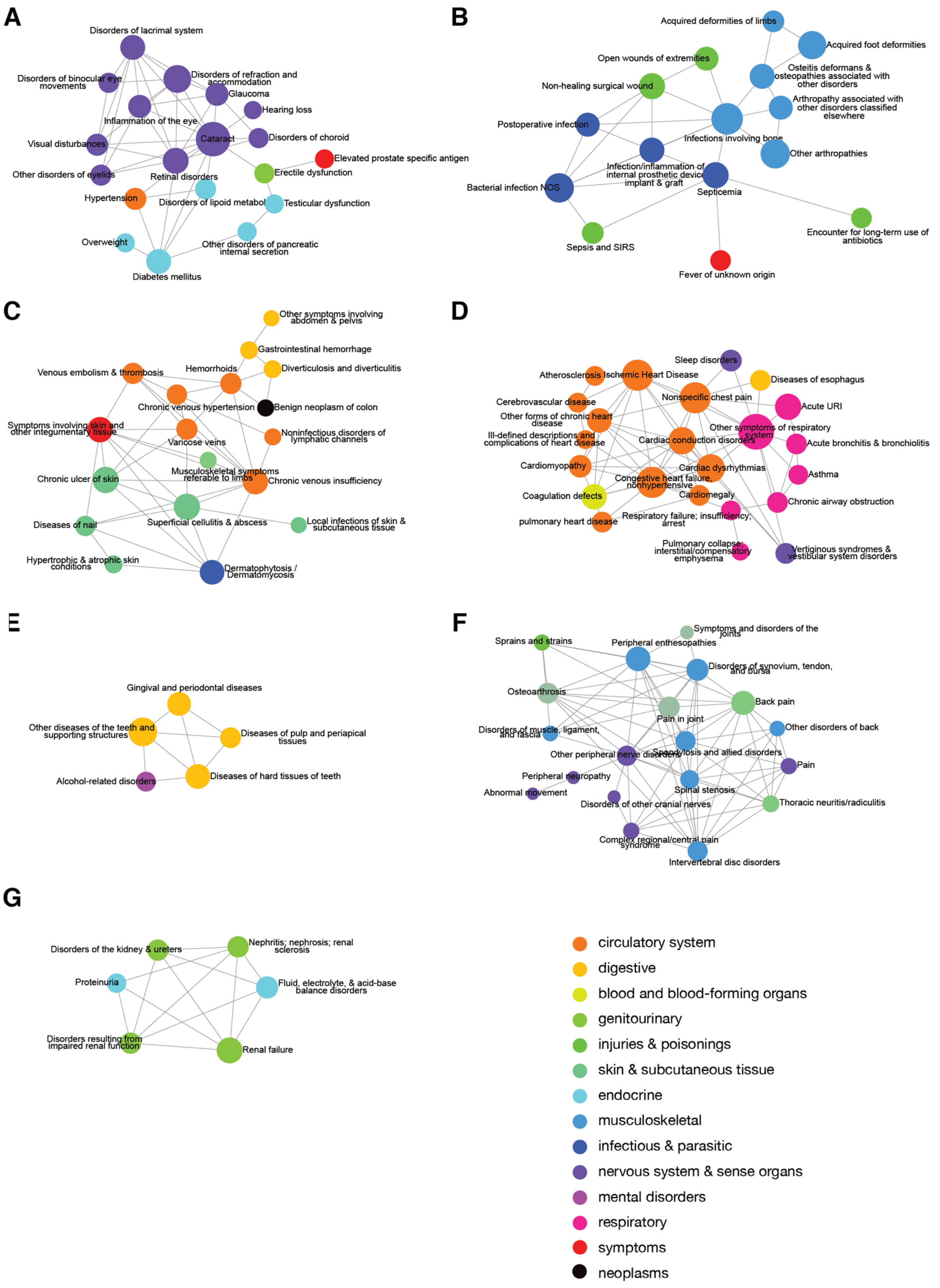

Supplementary Note

Study Populations

MVP Cohort: The design of the MVP has been previously described.[1] Briefly, individuals aged 19 to 104 years with the mean age of 62 years have been recruited from over 60 Veterans Health Administration medical centers nationwide since 2011. Each veteran's EHR is being integrated into the MVP biorepository, including inpatient International Classification of Diseases (ICD9/10) diagnosis codes, Current Procedural Terminology (CPT) procedure codes, clinical laboratory measurements, and reports of diagnostic imaging modalities. MVP has received ethical and study protocol approval by the VA Central Institutional Review Board in accordance with the principles outlined in the Declaration of Helsinki.

UK Biobank: UK Biobank is one of the largest and a detailed prospective study with over 500,000 participants aged 40–69 years recruited in 2006–2010. [2] The UK Biobank design including access to genetic data is well described in published reports[2] and in a publicly available website (see UK Biobank). The study has collected extensive phenotypic and genotypic data about its participants, including questionnaires, physical measures, sample assays, accelerometry, multimodal imaging, genome-wide genotyping and longitudinal follow-up for a wide range of health-related outcomes. We used UK Biobank only GWAS summary statistics from a recent UK Biobank GWAS for BMI that was included in a meta-analysis of the GIANT Consortium and UK Biobank BMI.[3]

GIANT Consortium: The Genetic Investigation of Anthropometric Traits (GIANT) consortium is an international collaboration that seeks to identify genetic loci that modulate human body size and shape, including height and measures of obesity. We have obtained the summary statistics of the most recent BMI-GWAS results from the GIANT consortium as evidence for replication. [4,5] As previously described,[3] the GIANT GWAS meta-analysis for common variants consisted of a two-stage meta-analysis to identify BMI-associated loci in adults of European ancestry. Stage 1 of the meta-analysis was performed across 80 GWAS studies (n=234,069) and stage 2 used data from 34 additional GWAS studies (n=88,137) genotyped using Metabochip7. Fixed effects meta-analyses were conducted using the inverse variance-weighted method implemented in METAL. All contributing GWAS common SNPs were imputed using the HapMap phase II CEU reference panel for European-descent studies. Study-specific GWAS results as well as GWAS meta-analysis results were corrected for genomic control.

The AAAGC: To replicate the results from the MVP for African ancestry, we have obtained the summary results from the most recent and largest African ancestry-based BMI-GWAS published by the African Ancestry Anthropometry Genetics Consortium (AAAGC). [6] For the AAAGC analysis,[5] a three-stage design was used to evaluate genetic associations with BMI. Stage 1 included GWAS meta-analyses in AA individuals and stage 2 included replication of top associations from stage 1; stage 3 included meta-analysis of top associations from stages 1 and 2 AA studies. The discovery stage 1 of AAAGC used 17 GWAS studies of up to n=42,752 AA individuals for BMI analyses. Stage 2 replication for BMI was performed in an additional n=10,143 AA individuals from AAAGC followed by meta-analysis with EA individuals from the GIANT

consortium (n=322,154 for BMI). Contributing SNPs were imputed using 1000 Genomes imputation.

Quality Control Analysis and Imputation
DNA extracted from participants' blood was genotyped using a customized Affymetrix Axiom® biobank array, the MVP 1.0 Genotyping Array. The array was enriched for both common and rare genetic variants of clinical significance in different ancestral backgrounds. Quality-control procedures used to assign ancestry, remove low-quality samples and variants, and perform genotype imputation were previously described[7] and briefly summarized. We excluded: duplicate samples, samples with more heterozygosity than expected, an excess (>2.5%) of missing genotype calls, or discordance between genetically inferred sex and phenotypic gender.[7] In addition, one individual from each pair of related individuals (more than second degree relatedness as measured by the KING[8] software) were removed. Prior to imputation, variants that were poorly called or that deviated from their expected allele frequency based on reference data from the 1000 Genomes Project[9] were excluded. After pre-phasing using EAGLE[10] v2, genotypes from the 1000 Genomes Project[9] phase 3, version 5 reference panel were imputed into Million Veteran Program (MVP) participants via Minimac3 software[11]. Global and ancestry-specific principal component analysis (PCA) was performed using the flashPCA software. Principal component analysis plots of all MVP participants is provided in Supplementary Figure 1 to present the genetic heterogeneity of the MVP participants and relative homogeneity within European ancestry and African ancestry groups (see the definition in the next section).
Following imputation, variant level quality control was performed using the EasyQC R package [12] (www.R-project.org), and exclusion metrics included: ancestry specific Hardy-Weinberg equilibrium[13] *p-value* $<1x10^{-20}$, posterior call probability < 0.9, imputation quality <0.3, minor allele frequency (MAF) < 0.0003, call rate < 97.5% for common variants (MAF > 1%), and call rate < 99% for rare variants (MAF < 1%). Variants were also excluded if they deviated > 10% from their expected allele frequency based on reference data from the 1000 Genomes Project[9].

Admixture analysis

We first extracted ancestry information from both self-reported and inferred sources. For self-reported ancestry, we extracted information on self-reported ethnicity and race from questions 4 and 5, respectively, from the MVP baseline survey administered to participants as a part of enrollment. For genetically-inferred ancestry, we ran the program ADMIXTURE [14] in the supervised mode using five sub-populations from the 1000 Genomes Phase 3 dataset [9] . The five reference populations are: CHB for Han Chinese in Beijing, GBR for British in England and Scotland, LWK for Luhya in Webuye, PEL Peruvians from Lima, YRI Yoruba in Ibadan, Nigeria. They represent East Asia, Europe, Eastern Africa, America, and Western Africa, respectively. We compared groups of individuals self-identifying as "White" or "White, non-Hispanic" to the fraction of their ancestry that aligned with the GBR reference populations. On top of the phenotypic definition, we retained samples with > 50% of their genome aligning with GBR for genetically European ancestry and samples with > 50% of their genome aligning with LWK or YRI for genetically African ancestry.

Functional Analysis of BMI-associated SNPs

We used FUMA (http://fuma.ctglab.nl/) to analyze the functional relevance of the novel loci identified from the combined meta-analysis as well as variants that are in high linkage disequilibrium with the lead SNPs ($r^2 > 0.6$, $p<1\times 10^{-5}$). Genome-wide significant SNPs ($p < 5 \times10^{-8}$) were grouped into a genomic locus if they were not independent from each other at r2 > 0.1 or physically close (distance < 500kb). Lead SNPs were defined within each locus if they were independent ($r^2 < 0.1$) and genome-wide significant. For non-Hispanic European ancestry, over 1000 protein-coding genes were positional mapped by the novel loci, and the top mapped genes include *UNC79*, *COX8C*, *UBN1*, *PPL*, *ABHD17A* and *PLEKHJ1*. Most of the variants were annotated as intronic (42.9%) or intergenic (39.3%), while 1%, 0.46% and 1.3% were annotated as exonic, at 5' UTR and at 3' UTR, respectively. Further exploration of the functional consequences of mapped exonic variants has prioritized 75 genes with non-synonymous variations. We also performed eQTL mapping based on 48 tissue types from GTEx V7. 611 genes were mapped to eQTLs (FDR q<0.05) and the top tissues involved were tibial nerve, thyroid and skeletal muscle. Differentially expressed gene enrichment analyses have shown significant (Bonferroni corrected p<0.05) up-regulation for thyroid, pituitary, brain and prostate tissues, and down-regulation for heart, liver, adrenal gland, muscle and blood tissues. Pathway analyses curated from Gene Ontology have revealed top associations with neurogenesis, behavior and carbohydrate derivative metabolic process. For non-Hispanic African ancestry, the topped positional mapped genes for the 6 novel loci were *RAI1, SREBF1, MOB1B* and *DCK*. No exonic variants were identified. 12 genes (FDR q<0.05) were identified through eQTL mapping, thyroid, esophagus mucosa, skeletal muscle and tibial nerve tissues were mapped with the strongest associations. Differentially expressed gene enrichment analyses have shown significant (Bonferroni corrected p<0.05) down-regulation for pancreas and small intestine tissues. Top biological functions identified from the pathway analysis include regulation of cell polarity, regulation of protein targeting to mitochondrion, circadian rhythm and regulation of intracellular protein transport.

PheWAS Quality Control, Disease Definitions, and Association Analysis

Of 353,323 genotyped veterans, participants were included in the phenome-wide analysis if the electronic health record reflected 2 or more separate encounters in the VA Healthcare System in each of the two years prior to enrollment in MVP. We included 21,209,658 prevalent diagnosis codes in the PheWAS analysis. We focused on the European ancestry, in which the mean age was 63.95 ± 13.11 years, and 93.0% were male.

Diagnosis codes were collapsed to clinical disease groups and corresponding controls using the groupings proposed by Denny et al [15]. Diseases were required to have a prevalence of ≥ 200 cases and 200 controls to be included in the phenome-wide analysis. Each polygenic risk score (PRS) was tested using logistic regression adjusting for age, sex, and ten principal components using the PheWAS R package (https://github.com/PheWAS/PheWAS) in R v3.2.0 (www.R-project.org). In total, 1,244

and 833 disease phenotypes (phecodes) were available for analysis in 174,531 EA and 49,695 AA participants, respectively.

We used the same set of phecodes included in the PheWAS[15] and therefore applied the same threshold for significance. We first ran linear regression with BMI as the dependent variable and GRS_{BMI} as the independent variable to calculate genetically instrumented BMI, then logistic regression was conducted to estimate the association between genetically instrumented BMI and phecodes. Genetically instrumented BMI was standardized to the scale of raw BMI to compare the effect from the MR analysis with associations obtained from observational studies. Thus, all odds ratios (ORs) were scaled by per standard deviation (SD) of BMI. We controlled for age, sex and principle components in both stages.[16] Because the sample size was larger and the genetic instrument (i.e., GRS_{BMI}) was stronger in the EA sample than in the AA sample, we present the phenome-wide MR results in EAs as our primary analysis.

Results for PheWAS and Network analysis

An additional 197 phecodes from 16 disease systems were associated with BMI but were not associated ($p>0.05$) with genetically influenced BMI (Supplementary Table 6). Of these BMI-associated phecodes, 60.4% (n=119) were noted to be inversely associated with BMI (Supplementary Table 6). The finding of a non-significant association in the GRS_{BMI}-based MR is inconsistent with a significant increased risk or protective role of BMI but may suggest reverse causality. As there was no prior published evidence for many of the MR associations, many of our MR findings in individuals of European ancestry are novel.

We identified seven disease communities, which were comprised of diseases from multiple disease systems (e.g., Community A: circulatory, endocrine, nervous systems, genitourinary, and general symptoms). In addition, some communities are dominated by closed related clinical conditions. Community A (Supplementary Figure 7A) included 11 disease codes related to sensory organs, but mostly related to eye disorders such as cataract, glaucoma, inflammation of the eye, and other retinal disorders. Community C contains vascular diseases and skin diseases (Supplementary Figure 7C). Community D includes mostly heart disease codes such as ischemic heart disease, congestive heart failure and chest pain. Community G includes renal diseases such as renal failure, nephritis and proteinuria.

Morbid obesity, associated with an increased burden of comorbid conditions in our study, is well known to confer increased risk for morbidity and mortality in hospitalized patients, and obesity is prevalent in patients hospitalized with acute illness including COVID-19 patients,[17] in whom acute respiratory failure, cardiac failure and renal failure are common.

To evaluate the impact of bi-directional Mendelian randomization (MR) of traits reported in our MR PheWAS studies of BMI, we selected 10 traits across a broad range of disease areas for which GWAS summary data has been curated and centralized by the Medical

Research Council Integrative Epidemiology Unit (MRC-IEU) open GWAS database (https://gwas.mrcieu.ac.uk). The list of the 10 traits is provided in the Table below. We used the BMI GWAS published by the GIANT consortium in 2015 (GWAS ID: ieu-a-2), which is widely used as a global reference for many exemplary analyses including MR. This GWAS does not use samples overlapping with UK Biobank so as to avoid potential bias in estimating causal effects. We used R package 'TwoSampleMR' to run the bi-directional MR. This package by default applied clumping to identify independent genetic variants for each individual GWAS summary data. Clumps are formed around index variants with P-value < 5e-8. Unsurprisingly, there is a strong bidirectional (two-way) "causal" effect between body mass index and type 2 diabetes. However, none of the other nine traits demonstrated evidence of a significant bidirectional effect to BMI, defined as inverse variance weighted (IVW) -based MR P-value < 0.005 (corrected for 10 selected traits). We recognize that a more systematic bi-directional MR analysis would be needed to evaluate a greater set of traits and our analysis is preliminary. For example, we did not include sensitivity analysis using Steiger filtering.[18] However, we believe our analysis provides an exploratory framework for assuming that significant bidirectional MR may be uncommon. We note that there is considerable inconsistency of available genetic instruments (i.e., genetic risk scores) for many conditions in PheWAS. A more comprehensive analysis is being pursed in other, future research projects, and would be beyond the scope of our current project.

Supplementary Table 1. Bi-directional MR analysis for ten selected traits across a range of disease areas

GWAS ID	Year	Trait	Sample size
ukb-d-I9_HEARTFAIL_NS	2018	Heart failure, not strict	361,194
ukb-b-7582	2018	Essential hypertension	462,933
ukb-b-12040	2018	Deep venous thrombosis	462,933
ukb-b-964	2018	Atrial fibrillation	463,010
ukb-b-10537	2018	Psoriasis	462,933
ukb-b-18700	2018	Cholelithiasis	462,933
ukb-b-13806	2018	Type 2 diabetes	462,933
ukb-b-12765	2018	Gout	463,010
ukb-b-20208	2018	Asthma	463,010
ukb-b-17194	2018	Macular degeneration	150,642

VA Million Veteran Program: Core Acknowledgement for Publications
Updated May 26, 2020

MVP Executive Committee

- Co-Chair: J. Michael Gaziano, M.D., M.P.H.
  VA Boston Healthcare System, 150 S. Huntington Avenue, Boston, MA 02130
- Co-Chair: Sumitra Muralidhar, Ph.D.
  US Department of Veterans Affairs, 810 Vermont Avenue NW, Washington, DC 20420
- Rachel Ramoni, D.M.D., Sc.D., Chief VA Research and Development Officer
  US Department of Veterans Affairs, 810 Vermont Avenue NW, Washington, DC 20420
- Jean Beckham, Ph.D.
  Durham VA Medical Center, 508 Fulton Street, Durham, NC 27705
- Kyong-Mi Chang, M.D.
  Philadelphia VA Medical Center, 3900 Woodland Avenue, Philadelphia, PA 19104
- Christopher J. O'Donnell, M.D., M.P.H.
  VA Boston Healthcare System, 150 S. Huntington Avenue, Boston, MA 02130
- Philip S. Tsao, Ph.D.
  VA Palo Alto Health Care System, 3801 Miranda Avenue, Palo Alto, CA 94304
- James Breeling, M.D., Ex-Officio
  US Department of Veterans Affairs, 810 Vermont Avenue NW, Washington, DC 20420
- Grant Huang, Ph.D., Ex-Officio
  US Department of Veterans Affairs, 810 Vermont Avenue NW, Washington, DC 20420
- JP Casas Romero, M.D., Ph.D., Ex-Officio
  VA Boston Healthcare System, 150 S. Huntington Avenue, Boston, MA 02130

MVP Program Office

- Sumitra Muralidhar, Ph.D.
  US Department of Veterans Affairs, 810 Vermont Avenue NW, Washington, DC 20420
- Jennifer Moser, Ph.D.
  US Department of Veterans Affairs, 810 Vermont Avenue NW, Washington, DC 20420

MVP Recruitment/Enrollment

- Recruitment/Enrollment Director/Deputy Director, Boston – Stacey B. Whitbourne, Ph.D.; Jessica V. Brewer, M.P.H.
  VA Boston Healthcare System, 150 S. Huntington Avenue, Boston, MA 02130
- MVP Coordinating Centers
  - Clinical Epidemiology Research Center (CERC), West Haven – Mihaela Aslan, Ph.D.

West Haven VA Medical Center, 950 Campbell Avenue, West Haven, CT 06516

  - Cooperative Studies Program Clinical Research Pharmacy Coordinating Center, Albuquerque – Todd Connor, Pharm.D.; Dean P. Argyres, B.S., M.S.
    New Mexico VA Health Care System, 1501 San Pedro Drive SE, Albuquerque, NM 87108
  - Genomics Coordinating Center, Palo Alto – Philip S. Tsao, Ph.D.
    VA Palo Alto Health Care System, 3801 Miranda Avenue, Palo Alto, CA 94304
  - MVP Boston Coordinating Center, Boston - J. Michael Gaziano, M.D., M.P.H.
    VA Boston Healthcare System, 150 S. Huntington Avenue, Boston, MA 02130
  - MVP Information Center, Canandaigua – Brady Stephens, M.S.
    Canandaigua VA Medical Center, 400 Fort Hill Avenue, Canandaigua, NY 14424

- VA Central Biorepository, Boston – Mary T. Brophy M.D., M.P.H.; Donald E. Humphries, Ph.D.; Luis E. Selva, Ph.D.
  VA Boston Healthcare System, 150 S. Huntington Avenue, Boston, MA 02130
- MVP Informatics, Boston – Nhan Do, M.D.; Shahpoor Shayan
  VA Boston Healthcare System, 150 S. Huntington Avenue, Boston, MA 02130
- MVP Data Operations/Analytics, Boston – Kelly Cho, Ph.D.
  VA Boston Healthcare System, 150 S. Huntington Avenue, Boston, MA 02130

MVP Science

- Science Operations – Christopher J. O'Donnell, M.D., M.P.H.
  VA Boston Healthcare System, 150 S. Huntington Avenue, Boston, MA 02130
- Genomics Core - Christopher J. O'Donnell, M.D., M.P.H.; Saiju Pyarajan Ph.D.
  VA Boston Healthcare System, 150 S. Huntington Avenue, Boston, MA 02130
  Philip S. Tsao, Ph.D.
  VA Palo Alto Health Care System, 3801 Miranda Avenue, Palo Alto, CA 94304
- Phenomics Core- Kelly Cho, M.P.H, Ph.D.
  VA Boston Healthcare System, 150 S. Huntington Avenue, Boston, MA 02130
- Data and Computational Sciences – Saiju Pyarajan, Ph.D.
  VA Boston Healthcare System, 150 S. Huntington Avenue, Boston, MA 02130
- Statistical Genetics – Elizabeth Hauser, Ph.D.
  Durham VA Medical Center, 508 Fulton Street, Durham, NC 27705
  Yan Sun, Ph.D.
  Atlanta VA Medical Center, 1670 Clairmont Road, Decatur, GA 30033
  Hongyu Zhao, Ph.D.
  West Haven VA Medical Center, 950 Campbell Avenue, West Haven, CT 06516

Current MVP Local Site Investigators

- Atlanta VA Medical Center (Peter Wilson, M.D.)
  1670 Clairmont Road, Decatur, GA 30033
- Bay Pines VA Healthcare System (Rachel McArdle, Ph.D.)
  10,000 Bay Pines Blvd Bay Pines, FL 33744
- Birmingham VA Medical Center (Louis Dellitalia, M.D.)
  700 S. 19th Street, Birmingham AL 35233
- Central Western Massachusetts Healthcare System (Kristin Mattocks, Ph.D., M.P.H.)
  421 North Main Street, Leeds, MA 01053
- Cincinnati VA Medical Center (John Harley, M.D., Ph.D.)
  3200 Vine Street, Cincinnati, OH 45220
- Clement J. Zablocki VA Medical Center (Jeffrey Whittle, M.D., M.P.H.)
  5000 West National Avenue, Milwaukee, WI 53295
- VA Northeast Ohio Healthcare System (Frank Jacono, M.D.)
  10701 East Boulevard, Cleveland, OH 44106
- Durham VA Medical Center (Jean Beckham, Ph.D.)
  508 Fulton Street, Durham, NC 27705
- Edith Nourse Rogers Memorial Veterans Hospital (John Wells., Ph.D.)
  200 Springs Road, Bedford, MA 01730
- Edward Hines, Jr. VA Medical Center (Salvador Gutierrez, M.D.)
  5000 South 5th Avenue, Hines, IL 60141
- Veterans Health Care System of the Ozarks (Gretchen Gibson, D.D.S., M.P.H.)
  1100 North College Avenue, Fayetteville, AR 72703
- Fargo VA Health Care System (Kimberly Hammer, Ph.D.)
  2101 N. Elm, Fargo, ND 58102
- VA Health Care Upstate New York (Laurence Kaminsky, Ph.D.)
  113 Holland Avenue, Albany, NY 12208
- New Mexico VA Health Care System (Gerardo Villareal, M.D.)
  1501 San Pedro Drive, S.E. Albuquerque, NM 87108
- VA Boston Healthcare System (Scott Kinlay, M.B.B.S., Ph.D.)
  150 S. Huntington Avenue, Boston, MA 02130
- VA Western New York Healthcare System (Junzhe Xu, M.D.)
  3495 Bailey Avenue, Buffalo, NY 14215-1199
- Ralph H. Johnson VA Medical Center (Mark Hamner, M.D.)
  109 Bee Street, Mental Health Research, Charleston, SC 29401
- Columbia VA Health Care System (Roy Mathew, M.D.)
  6439 Garners Ferry Road, Columbia, SC 29209
- VA North Texas Health Care System (Sujata Bhushan, M.D.)
  4500 S. Lancaster Road, Dallas, TX 75216
- Hampton VA Medical Center (Pran Iruvanti, D.O., Ph.D.)
  100 Emancipation Drive, Hampton, VA 23667
- Richmond VA Medical Center (Michael Godschalk, M.D.)

1201 Broad Rock Blvd., Richmond, VA 23249

- Iowa City VA Health Care System (Zuhair Ballas, M.D.)
  601 Highway 6 West, Iowa City, IA 52246-2208
- Eastern Oklahoma VA Health Care System (Douglas Ivins, M.D.)
  1011 Honor Heights Drive, Muskogee, OK 74401
- James A. Haley Veterans' Hospital (Stephen Mastorides, M.D.)
  13000 Bruce B. Downs Blvd, Tampa, FL 33612
- James H. Quillen VA Medical Center (Jonathan Moorman, M.D., Ph.D.)
  Corner of Lamont & Veterans Way, Mountain Home, TN 37684
- John D. Dingell VA Medical Center (Saib Gappy, M.D.)
  4646 John R Street, Detroit, MI 48201
- Louisville VA Medical Center (Jon Klein, M.D., Ph.D.)
  800 Zorn Avenue, Louisville, KY 40206
- Manchester VA Medical Center (Nora Ratcliffe, M.D.)
  718 Smyth Road, Manchester, NH 03104
- Miami VA Health Care System (Hermes Florez, M.D., Ph.D.)
  1201 NW 16th Street, 11 GRC, Miami FL 33125
- Michael E. DeBakey VA Medical Center (Olaoluwa Okusaga, M.D.)
  2002 Holcombe Blvd, Houston, TX 77030
- Minneapolis VA Health Care System (Maureen Murdoch, M.D., M.P.H.)
  One Veterans Drive, Minneapolis, MN 55417
- N. FL/S. GA Veterans Health System (Peruvemba Sriram, M.D.)
  1601 SW Archer Road, Gainesville, FL 32608
- Northport VA Medical Center (Shing Shing Yeh, Ph.D., M.D.)
  79 Middleville Road, Northport, NY 11768
- Overton Brooks VA Medical Center (Neeraj Tandon, M.D.)
  510 East Stoner Ave, Shreveport, LA 71101
- Philadelphia VA Medical Center (Darshana Jhala, M.D.)
  3900 Woodland Avenue, Philadelphia, PA 19104
- Phoenix VA Health Care System (Samuel Aguayo, M.D.)
  650 E. Indian School Road, Phoenix, AZ 85012
- Portland VA Medical Center (David Cohen, M.D.)
  3710 SW U.S. Veterans Hospital Road, Portland, OR 97239
- Providence VA Medical Center (Satish Sharma, M.D.)
  830 Chalkstone Avenue, Providence, RI 02908
- Richard Roudebush VA Medical Center (Suthat Liangpunsakul, M.D., M.P.H.)
  1481 West 10th Street, Indianapolis, IN 46202
- Salem VA Medical Center (Kris Ann Oursler, M.D.)
  1970 Roanoke Blvd, Salem, VA 24153
- San Francisco VA Health Care System (Mary Whooley, M.D.)
  4150 Clement Street, San Francisco, CA 94121
- South Texas Veterans Health Care System (Sunil Ahuja, M.D.)
  7400 Merton Minter Boulevard, San Antonio, TX 78229

- Southeast Louisiana Veterans Health Care System (Joseph Constans, Ph.D.)
  2400 Canal Street, New Orleans, LA 70119
- Southern Arizona VA Health Care System (Paul Meyer, M.D., Ph.D.)
  3601 S 6th Avenue, Tucson, AZ 85723
- Sioux Falls VA Health Care System (Jennifer Greco, M.D.)
  2501 W 22nd Street, Sioux Falls, SD 57105
- St. Louis VA Health Care System (Michael Rauchman, M.D.)
  915 North Grand Blvd, St. Louis, MO 63106
- Syracuse VA Medical Center (Richard Servatius, Ph.D.)
  800 Irving Avenue, Syracuse, NY 13210
- VA Eastern Kansas Health Care System (Melinda Gaddy, Ph.D.)
  4101 S 4th Street Trafficway, Leavenworth, KS 66048
- VA Greater Los Angeles Health Care System (Agnes Wallbom, M.D., M.S.)
  11301 Wilshire Blvd, Los Angeles, CA 90073
- VA Long Beach Healthcare System (Timothy Morgan, M.D.)
  5901 East 7th Street Long Beach, CA 90822
- VA Maine Healthcare System (Todd Stapley, D.O.)
  1 VA Center, Augusta, ME 04330
- VA New York Harbor Healthcare System (Scott Sherman, M.D., M.P.H.)
  423 East 23rd Street, New York, NY 10010
- VA Pacific Islands Health Care System (George Ross, M.D.)
  459 Patterson Rd, Honolulu, HI 96819
- VA Palo Alto Health Care System (Philip Tsao, Ph.D.)
  3801 Miranda Avenue, Palo Alto, CA 94304-1290
- VA Pittsburgh Health Care System (Patrick Strollo, Jr., M.D.)
  University Drive, Pittsburgh, PA 15240
- VA Puget Sound Health Care System (Edward Boyko, M.D.)
  1660 S. Columbian Way, Seattle, WA 98108-1597
- VA Salt Lake City Health Care System (Laurence Meyer, M.D., Ph.D.)
  500 Foothill Drive, Salt Lake City, UT 84148
- VA San Diego Healthcare System (Samir Gupta, M.D., M.S.C.S.)
  3350 La Jolla Village Drive, San Diego, CA 92161
- VA Sierra Nevada Health Care System (Mostaqul Huq, Pharm.D., Ph.D.)
  975 Kirman Avenue, Reno, NV 89502
- VA Southern Nevada Healthcare System (Joseph Fayad, M.D.)
  6900 North Pecos Road, North Las Vegas, NV 89086
- VA Tennessee Valley Healthcare System (Adriana Hung, M.D., M.P.H.)
  1310 24th Avenue, South Nashville, TN 37212
- Washington DC VA Medical Center (Jack Lichy, M.D., Ph.D.)
  50 Irving St, Washington, D. C. 20422
- W.G. (Bill) Hefner VA Medical Center (Robin Hurley, M.D.)
  1601 Brenner Ave, Salisbury, NC 28144
- White River Junction VA Medical Center (Brooks Robey, M.D.)

163 Veterans Drive, White River Junction, VT 05009

- William S. Middleton Memorial Veterans Hospital (Robert Striker, M.D., Ph.D.)
  2500 Overlook Terrace, Madison, WI 53705

Supplementary References

1. Gaziano JM, Concato J, Brophy M, et al. Million Veteran Program: a mega-biobank to study genetic influences on health and disease. *Journal of Clinical Epidemiology* 70:214-23 (2016).
2. Sudlow C, Gallacher J, Allen N, et al. UK biobank: an open access resource for identifying the causes of a wide range of complex diseases of middle and old age. *PLoS Medicine* 12:e1001779 (2015).
3. Yengo L, Sidorenko J, Kemper KE, et al. Meta-analysis of genome-wide association studies for height and body mass index in approximately 700000 individuals of European ancestry. *Hum Mol Genet* 27:3641-9 (2018).
4. Locke AE, Kahali B, Berndt SI, et al. Genetic studies of body mass index yield new insights for obesity biology. *Nature* 518:197 (2015).
5. Turcot V, Lu Y, Highland HM, et al. Protein-altering variants associated with body mass index implicate pathways that control energy intake and expenditure in obesity. *Nature Genetics* 50:26 (2018).
6. Ng MC, Graff M, Lu Y, et al. Discovery and fine-mapping of adiposity loci using high density imputation of genome-wide association studies in individuals of African ancestry: African Ancestry Anthropometry Genetics Consortium. *PLoS Genetics* 13:e1006719 (2017).
7. Hunter-Zinck H, Shi Y, Li M, et al. Genotyping Array Design and Data Quality Control in the Million Veteran Program. *Am J Hum Genet* 106:535-48 (2020).
8. Manichaikul A, Mychaleckyj JC, Rich SS, Daly K, Sale M, Chen WM. Robust relationship inference in genome-wide association studies. *Bioinformatics* 26:2867-73 (2010).
9. Genomes Project C, Auton A, Brooks LD, et al. A global reference for human genetic variation. *Nature* 526:68-74 (2015).
10. Loh PR, Palamara PF, Price AL. Fast and accurate long-range phasing in a UK Biobank cohort. *Nature Genetics* 48:811-6 (2016).
11. Howie B, Marchini J, Stephens M. Genotype imputation with thousands of genomes. G3 (Bethesda) 1:457-70 (2011).
12. Winkler TW, Day FR, Croteau-Chonka DC, et al. Quality control and conduct of genome-wide association meta-analyses. *Nature Protocols* 9:1192-212 (2014).
13. Hyde CL, Nagle MW, Tian C, et al. Identification of 15 genetic loci associated with risk of major depression in individuals of European descent. *Nature Genetics* 48:1031-6 (2016).
14. Alexander DH, Novembre J, Lange K. Fast model-based estimation of ancestry in unrelated individuals. *Genome Res* 19:1655-64 (2009).
15. Denny JC, Bastarache L, Ritchie MD, et al. Systematic comparison of phenome-wide association study of electronic medical record data and genome-wide association study data. *Nature Biotechnology* 31:1102-10 (2013).
16. Burgess S, Small DS, Thompson SG. A review of instrumental variable estimators for Mendelian randomization. *Stat Methods Med Res* 26:2333-55 (2017).
17. Richardson S, Hirsch JS, Narasimhan M, et al. Presenting Characteristics, Comorbidities, and Outcomes Among 5700 Patients Hospitalized With COVID-19 in the New York City Area. *JAMA* 323:2052-2059 (2020).

18. Hemani G, Tilling K, Davey Smith G. Orienting the causal relationship between imprecisely measured traits using GWAS summary data. *PLoS Genetics* 13:e1007081 (2017).
