# Supplementary material for: Genomics and phenomics of body mass index reveals a complex disease network
Source: Nat Commun. 2022 Dec 29;13:7973. doi: 10.1038/s41467-022-35553-2 (PMC9798356; doi:10.1038/s41467-022-35553-2)
Supplement: Supplementary file 2 — Description of Additional Supplementary Files [file 41467_2022_35553_MOESM2_ESM.pdf]

**Description of Additional Supplementary Files**

File Name: Supplementary Data 1
Description: Genome-wide significant loci in BMI meta-analysis of European ancestry combining the MVP, the UK Biobank, and the GIANT Consortium

File Name: Supplementary Data 2
Description: Genome-wide significant loci in BMI meta-analysis of African ancestry combining the MVP and the AAAGC consortium

File Name: Supplementary Data 3
Description: BMI associated diagnosis codes after Bonferroni correction. (N=661). ICD-code: International Classification of Diseases (ICD)-9 code; group: disease systems: N Cases: number of cases; N Controls: number of controls; OR: odds ratio; L95CI: lower bound of 95% confidence interval of the OR; U95CI: upper bound of 95% confidence interval of the OR.

File Name: Supplementary Data 4
Description: Significant associations in the BMI association analysis, and $PRS_{BMI}$-based Mendelian Randomization (MR) analysis in the MVP participants of European ancestry. Group: disease systems: N Cases: number of cases; N Controls: number of controls; OR: odds ratio; L95CI: lower bound of 95% confidence interval of the OR; U95CI: upper bound of 95% confidence interval of the OR. The effects were standardized by standard deviation (SD) of body mass index (BMI).

File Name: Supplementary Data 5
Description: Summary of two-sample MR analyses (inverse-variance weighted, median weighted and MR Egger) of significant phecodes in the $PRS_{BMI}$-based phenome-wide Mendelian Randomization (MR) analysis. Group: disease systems; N Cases: number of cases; N Controls: number of controls; OR: odds ratio. The effects were standardized by standard deviation (SD) of BMI.

File Name: Supplementary Data 6
Description: Burden of significant phecodes in the phenome-wide Mendelian Randomization (MR) analysis across obesity categories among EA participants.

File Name: Supplementary Data 7
Description: Disease codes significantly associated with BMI but insignificant (p>0.05) in the $GRS_{BMI}$-based Mendelian Randomization (MR) analysis. Group: disease systems; N Cases: number of cases; N Controls: number of controls; OR: odds ratio; L95CI: lower bound of 95% confidence interval of the OR; U95CI: upper bound of 95% confidence interval of the OR. The effects were standardized by standard deviation (SD) of BMI.

File Name: Supplementary Data 8
Description: Significant associations in the $GRS_{BMI}$-based Mendelian Randomization (MR) analysis in the MVP participants of African ancestry. Group: disease systems: N

Cases: number of cases; N Controls: number of controls; OR: odds ratio; L95CI: lower bound of 95% confidence interval of the OR; U95CI: upper bound of 95% confidence interval of the OR. The effects were standardized by standard deviation (SD) of body mass index (BMI).

File Name: Supplementary Data 9
Description: Network properties of Phenotypic Network Map. The table includes Code: 3-digit high-level diagnosis code; degree: network connectivity; $\sum\phi_i$: sum of phi-coefficients of all links connecting to node I (i.e., weighted degree); community: network community number assigned by the algorithm; statistical significance of network community; and the corresponding p-value.
