# Supplementary material for: Genomics and phenomics of body mass index reveals a complex disease network
Source: Nat Commun. 2022 Dec 29;13:7973. doi: 10.1038/s41467-022-35553-2 (PMC9798356; doi:10.1038/s41467-022-35553-2)
Supplement: Supplementary file 4 — Reporting Summary [file 41467_2022_35553_MOESM4_ESM.pdf]

nature portfolio

Corresponding author(s): O'Donnell, Sun

Last updated by author(s): Nov 15, 2022

Reporting Summary

Nature Portfolio wishes to improve the reproducibility of the work that we publish. This form provides structure for consistency and transparency in reporting. For further information on Nature Portfolio policies, see our Editorial Policies and the Editorial Policy Checklist.

Statistics

For all statistical analyses, confirm that the following items are present in the figure legend, table legend, main text, or Methods section.

n/a	Confirmed	
☐	☒	The exact sample size (*n*) for each experimental group/condition, given as a discrete number and unit of measurement
☐	☒	A statement on whether measurements were taken from distinct samples or whether the same sample was measured repeatedly
☐	☒	The statistical test(s) used AND whether they are one- or two-sided<br>*Only common tests should be described solely by name; describe more complex techniques in the Methods section.*
☐	☒	A description of all covariates tested
☐	☒	A description of any assumptions or corrections, such as tests of normality and adjustment for multiple comparisons
☐	☒	A full description of the statistical parameters including central tendency (e.g. means) or other basic estimates (e.g. regression coefficient) AND variation (e.g. standard deviation) or associated estimates of uncertainty (e.g. confidence intervals)
☐	☒	For null hypothesis testing, the test statistic (e.g. *F*, *t*, *r*) with confidence intervals, effect sizes, degrees of freedom and *P* value noted<br>*Give P values as exact values whenever suitable.*
☒	☐	For Bayesian analysis, information on the choice of priors and Markov chain Monte Carlo settings
☒	☐	For hierarchical and complex designs, identification of the appropriate level for tests and full reporting of outcomes
☐	☒	Estimates of effect sizes (e.g. Cohen's *d*, Pearson's *r*), indicating how they were calculated

Our web collection on statistics for biologists contains articles on many of the points above.

Software and code

Policy information about availability of computer code

Data collection	no software was used as the data used for analysis was collected centrally as part of a larger research program outside of the scope of this manuscript
Data analysis	R v4.0.2; SQL Server v17; plink2a; METAL; GWAMA v2.2.2; minimac; eagle; ADMIXTURE;

For manuscripts utilizing custom algorithms or software that are central to the research but not yet described in published literature, software must be made available to editors and reviewers. We strongly encourage code deposition in a community repository (e.g. GitHub). See the Nature Portfolio guidelines for submitting code & software for further information.

Data

Policy information about availability of data

All manuscripts must include a data availability statement. This statement should provide the following information, where applicable:

- Accession codes, unique identifiers, or web links for publicly available datasets
- A description of any restrictions on data availability
- For clinical datasets or third party data, please ensure that the statement adheres to our policy

Due to US Department of Veterans Affairs (VA) regulations and our ethics agreements, the individual-level data sets used for this study are not permitted to leave the Million Veteran Program (MVP) research environment and VA firewall. This limitation is consistent with other MVP studies based on VA data. However, the MVP data are made available to researchers with an approved VA and MVP study protocol. The full summary level association results from genome-wide association

analyses in the MVP and the meta-analysis from this report are available through a standard application to dbGaP (accession number phs001672 [https://www.ncbi.nlm.nih.gov/projects/gap/cgi-bin/study.cgi?study_id=phs001672.v8.p1]). The only restriction is that use of the data is limited to health/medical/biomedical purposes, and does not include the study of population origins or ancestry. Use of the data does include methods development research (e.g., development and testing of software or algorithms) and requestors agree to make the results of studies using the data available to the larger scientific community.
Summary statistics of BMI GWAS from the GIANT and UK Biobank meta-analysis are available from https://portals.broadinstitute.org/collaboration/giant/index.php/GIANT_consortium_data_files
Summary association statistics from AAAGC BMI meta-analyses are available from dbGaP at accession number phs000930 [https://www.ncbi.nlm.nih.gov/projects/gap/cgi-bin/study.cgi?study_id=phs000930.v9.p1]

Human research participants

Policy information about studies involving human research participants and Sex and Gender in Research.

Reporting on sex and gender	Biological sex was determined through the clinical records and confirmed by genetics. Sex-specific analyses were not performed but sex was included as a covariate in all statistical models. MVP is mainly male due to the composition of the US Veteran population but recruitment aimed to over sample for women so the analyzed population is 93% male.
Population characteristics	Sample sizes of 215,734 European descent (93% male, mean age 64.0 years) and 55,525 African descent (87.6% male, mean age 57.9 years) Veterans, with other baseline characteristics described in the Table 1. Details of cohort participants from the replication cohorts are described in the respective methods papers described under "Recruitment" below.
Recruitment	Million Veteran Program participants were recruited from random sampling of Veteran users of over 60 Veterans Health Administrations Medical Centers in the United States. Methods of recruitment and population characteristics are described in the MVP Methods paper (Journal of Clinical Epidemiology 2016;70:214-223). Replication cohorts included the UK Biobank and Genomic Investigation of Anthropometric Traits (GIANT, see Nature 2015;518:197-206) Consortium for European descent replication and the African Ancestry Anthropometry Genetics Consortium (AAAGC, see PLoS Genetics 2017;13:e1006719) for African descent replication.
Ethics oversight	MVP has received ethical and study protocol approval by the VA Central Institutional Review Board in accordance with the principles outlined in the Declaration of Helsinki. All participants have provided written informed consent. Participants in the UK Biobank and cohorts of the GIANT Consortium and the AAAGC Consortium have also given written informed consent according to protocols described in the respective methods publications.

Note that full information on the approval of the study protocol must also be provided in the manuscript.

Field-specific reporting

Please select the one below that is the best fit for your research. If you are not sure, read the appropriate sections before making your selection.

☒ Life sciences ☐ Behavioural & social sciences ☐ Ecological, evolutionary & environmental sciences

For a reference copy of the document with all sections, see nature.com/documents/nr-reporting-summary-flat.pdf

Life sciences study design

All studies must disclose on these points even when the disclosure is negative.

Sample size	Sample size was not per-determined. All available samples with phenotypic and genetic data are included in this observational study to maximize the statistical power of discovery.
Data exclusions	All available samples with both phenotypic and genetic data are included in this observational study.
Replication	We replicated the genetic associations identified in the Million Veteran Program (MVP) using external studies including the UK Biobank, GIANT and AAAGC.
Randomization	Not applicable. This is an observational association study.
Blinding	Not applicable. This is an observational association study.

Reporting for specific materials, systems and methods

We require information from authors about some types of materials, experimental systems and methods used in many studies. Here, indicate whether each material, system or method listed is relevant to your study. If you are not sure if a list item applies to your research, read the appropriate section before selecting a response.

Materials & experimental systems

n/a	Involved in the study
☒	☐ Antibodies
☒	☐ Eukaryotic cell lines
☒	☐ Palaeontology and archaeology
☒	☐ Animals and other organisms
☒	☐ Clinical data
☒	☐ Dual use research of concern

Methods

n/a	Involved in the study
☒	☐ ChIP-seq
☒	☐ Flow cytometry
☒	☐ MRI-based neuroimaging
